# Is Earlier Always Better? Early Versus Later Time-Restricted Eating, Body Composition, and Exercise Performance: A Narrative Review

**DOI:** 10.3390/nu18213603

**Published:** 2026-10-30

**Authors:** Zifu Yu, Takeshi Ueda, Pengcheng Zhou

**Affiliations:** 1 Graduate School of Humanities and Social Sciences, Hiroshima University, Higashihiroshima 739-8524, Japan; yuzifusang@gmail.com; 2 College of Physical Education, Minnan Normal University, Zhangzhou 363000, China; houtei_syuu@foxmail.com

**Keywords:** time-restricted eating, early time-restricted eating, delayed time-restricted eating, meal timing, chrononutrition, circadian rhythm, lean mass, resistance training, exercise performance

## Abstract

**Background:** Time-restricted eating (TRE) confines daily food intake to a consistent window that may occur early (eTRE) or later in the day (delayed TRE, dTRE). Whether timing differentially affects body composition and exercise performance, and whether exercise training modifies these effects, remains unclear. **Methods:** PubMed, Google Scholar, and reference lists were searched. Twenty-three intervention studies were narratively synthesized by timing comparison and exercise context, with study-level methodological appraisal. **Results:** Eight studies directly compared earlier and later windows. None favored the later window for body composition. Greater weight or fat loss with eTRE came from one crossover trial and two non-randomized comparisons, whereas the randomized parallel-group trials, including the largest, detected no difference. With structured training, TRE reduced fat mass more than training alone in four of six trials, regardless of window type; lean-mass comparisons were imprecise. No direct comparison detected timing differences in strength, muscular endurance, or aerobic outcomes, but estimates were imprecise. Greater sprint power after dTRE in one crossover trial may reflect test-day feeding, learning, or order effects. **Conclusions:** Earlier eating was not consistently better, but timing cannot be considered irrelevant. Trials crossing window timing with controlled energy intake and matched training are needed.

## 1. Introduction

Obesity is a major global public health concern. In 2022, approximately 2.5 billion adults were living with being overweight, including 890 million with obesity, and the global prevalence of adult obesity had more than doubled since 1990 [1,2]. Obesity contributes substantially to the global burden of disease and increases the risk of cardiovascular disease, type 2 diabetes, and other chronic conditions [3,4]. Continuous caloric restriction (CCR) can produce meaningful short-term weight loss, but sustaining a prescribed energy deficit is difficult, and weight regain is common [5,6]. Moreover, for individuals combining dietary change with exercise, the goal is often not weight loss per se but reducing fat mass while preserving lean mass and physical performance.

Time-restricted eating (TRE), a form of intermittent fasting, confines daily food intake to a consistent window, typically 4–10 h, with only water or non-caloric beverages permitted outside this window [7,8,9]. Because it does not require calorie counting, TRE may be easier to implement than CCR for some individuals, and shortening the eating window often reduces energy intake spontaneously, although the magnitude of this reduction varies [7,10]. Systematic reviews and meta-analyses indicate that TRE produces modest reductions in body weight and fat mass and may improve selected cardiometabolic markers [11,12,13], and some of these benefits have been attributed to aligning food intake with circadian rhythms [14,15]. Effects on lean mass are less consistent; in one controlled-feeding trial, lean mass decreased more with TRE than with a longer eating window [16].

The position of the eating window within the day may modify these effects. Early TRE (eTRE) concentrates food intake in the morning and early afternoon, whereas delayed TRE (dTRE) shifts it toward midday and evening. Because insulin sensitivity, glucose tolerance, and diet-induced thermogenesis are generally more favorable earlier in the biological day, eTRE has been proposed to confer greater metabolic and weight-management benefits [17,18,19,20]. Direct comparisons, however, have been inconsistent. Some trials reported greater reductions in body weight or body fat with eTRE [21,22], whereas others, including the largest trial in this review, found similar body-composition changes with earlier and later windows [23,24,25]. In addition, one of the trials favoring eTRE for body weight found a greater improvement in anaerobic power with dTRE [21]. These discrepancies suggest that the relative effects of eTRE and dTRE may depend on the outcome examined rather than following a uniform “earlier is better” pattern.

Exercise adds a further layer of complexity. Resistance and concurrent training provide a strong independent stimulus for preserving lean mass and improving performance, and the interval between food intake and training may affect energy availability and recovery [26]. Recent meta-analyses suggest that TRE combined with exercise may modestly reduce fat mass without significant loss of fat-free mass or performance [27,28], but these analyses treated TRE as a single intervention category. Conversely, reviews comparing early and later TRE have focused mainly on body weight and cardiometabolic outcomes [19,20]. Consequently, it remains unclear whether eTRE and dTRE differ in their effects on body composition and exercise performance, and whether concurrent training modifies these differences.

This narrative review, therefore, synthesizes intervention studies examining earlier and later TRE in relation to body composition and exercise performance, considering studies with and without concurrent structured exercise separately. It addresses three questions: (1) whether eTRE and dTRE differ in their effects on body weight, fat mass, and lean mass; (2) whether their comparative effects are consistent across strength, muscular endurance, aerobic, and anaerobic performance; and (3) which circadian, nutritional, and exercise-related factors may explain the observed findings. The aim is to clarify what the current evidence can and cannot support when selecting a TRE schedule and to identify priorities for future trials.

## 2. Methods

### 2.1. Review Design and Scope

This structured narrative review synthesized human intervention studies examining how the timing of time-restricted eating (TRE) affects body composition and exercise performance. The principal comparison was early TRE (eTRE) versus eating windows positioned later in the day. Because terminology for later schedules varies across studies, delayed TRE (dTRE) is used as an umbrella term in the synthesis, whereas the original labels, such as midday TRE (mTRE) and late TRE (lTRE), are retained when individual studies are described.

Studies without concurrent structured exercise were synthesized separately from those combining TRE with resistance, aerobic, or concurrent training, because exercise training independently influences fat mass, lean mass, and physical performance and may, therefore, modify or obscure the effect of eating-window timing. Studies were classified as including concurrent exercise training when participants performed structured training during the intervention, whether newly prescribed by the investigators or maintained as part of their habitual training.

### 2.2. Information Sources and Search Strategy

PubMed was searched using three conceptual blocks combined with “AND”, with related terms within each block combined with “OR”:

(1) TRE: “time-restricted eating” OR “time-restricted feeding” OR “time-restricted diet” OR “TRE” OR “TRF” OR “intermittent fasting” OR “16:8” OR “eating window”;

(2) timing: “early” OR “midday” OR “late” OR “delayed” OR “morning” OR “evening” OR “timing” OR “chrononutrition”;

(3) outcomes: “body composition” OR “fat mass” OR “lean mass” OR “muscle mass” OR “fat-free mass” OR “body fat” OR “exercise performance” OR “physical performance” OR “muscle strength” OR “muscular endurance” OR “aerobic capacity” OR “anaerobic power” OR “explosive performance”.

Reference lists of relevant reviews, particularly those comparing early and later TRE or examining TRE in exercising populations, and of eligible primary studies were also examined, and forward and backward citation tracking was applied to potentially eligible studies. In addition, the authors’ existing literature library, compiled during previous research on TRE, was screened for relevant records. This initial search included studies available up to 12 June 2026 and yielded 154 unique candidate records after duplicate removal (100 from PubMed and 54 from reference lists, citation tracking, and the existing library). The three blocks above constitute the complete PubMed search string.

An updated search was conducted during manuscript preparation, including a Google Scholar search on 18 June 2026. Because Google Scholar limits query length and does not support the full Boolean structure, 10 sub-queries derived from the same terms were used; the abbreviations “TRE” and “TRF” were omitted to avoid irrelevant matches, and the first 100 results of each sub-query, sorted by relevance, were screened. During revision, the Google Scholar search was repeated on 13 October 2026 with the same sub-queries and settings, and the number of records retrieved and screened for each sub-query was recorded (Table S1).

### 2.3. Eligibility Criteria

Studies were included in the primary evidence synthesis if they met all of the following criteria:

1. Original human intervention trial of TRE, defined as confining food intake to a recurring eating window of no more than approximately 10 h per day, with only water or non-caloric beverages permitted outside the window.
2. The timing, or allowable timing range, of the eating window was reported in sufficient detail to interpret the intervention; early, midday, delayed, late, self-selected, and individualized windows were all eligible.
3. Intervention duration of at least four weeks.
4. A parallel comparison group or a within-participant crossover comparison condition.
5. At least one body-composition outcome (e.g., fat mass, body fat percentage, fat-free or lean mass, muscle thickness, or regional adiposity) or exercise-performance outcome (e.g., strength, muscular endurance, aerobic endurance, anaerobic power, or explosive performance). Studies reporting body weight without a body-composition measure were eligible only if they also assessed exercise performance.
6. Adult participants, including healthy, physically active or trained adults and adults with overweight or obesity.
7. Publication in English.

Studies were excluded if any of the following applied:

1. The intervention was a form of intermittent fasting other than TRE, such as alternate-day fasting, the 5:2 diet, or Ramadan fasting involving daytime fluid restriction.
2. Participants had a clinical condition, such as type 2 diabetes, likely to dominate the metabolic or physical response to TRE.
3. The publication was an observational study, protocol, conference abstract without a corresponding full-text article, animal study, or review. Reviews and meta-analyses were retained as background sources and for reference-list searching but were not included in data extraction.

Because definitions vary across the literature, no universal clock-time threshold was imposed to classify eating windows. Classification was based on the terminology and protocol of the original investigators together with the reported start and end times: windows beginning in the morning and ending by the early-to-mid afternoon were treated as eTRE, whereas windows beginning around midday or extending into the evening were treated as dTRE. Midday schedules were identified separately when so labeled by the original study and were not assumed to be physiologically equivalent to late-evening schedules. When an investigator-assigned label and the reported clock times diverged, for example an “early” window defined relative to waking or ending in the late afternoon, the original label was retained and the actual eating times are reported in Table 1 and Table 2. Self-selected or individualized windows that could not be assigned to either category were analyzed as a separate evidence category rather than being forced into an early or late classification.

### 2.4. Study Selection

Records were screened by title and abstract, and full texts were examined when eligibility could not be determined from the abstract alone, to confirm the study design, intervention duration, eating-window timing, comparison condition, participant characteristics, and outcomes. Screening and full-text assessment were performed by one reviewer (Z.Y.), and full texts were available for all potentially eligible records.

When a conference abstract was subsequently published as a full article, only the full article was used. Multiple publications from the same cohort were identified by trial registration number, sample size, baseline characteristics, intervention protocol, authorship, and institutional affiliation, and were treated as a single study; the primary publication served as the main data source, and companion or follow-up publications were consulted for additional outcomes.

Of the 154 candidate records, 95 were excluded at the title and abstract stage because of an unrelated topic, an ineligible population or design, or a fasting protocol other than daily TRE (e.g., Ramadan fasting or the 5:2 diet), and 33 were retained as background literature. Sixteen records met all eligibility criteria on the basis of the title and abstract, which was confirmed during data extraction, whereas 10 records that could not be classified with sufficient confidence underwent full-text assessment. Of these 10 records, 5 were eligible, 4 were retained as narrative supplementary evidence because they did not meet one or more eligibility criteria (eating windows longer than 10 h, an intervention shorter than four weeks, or no comparison condition), and 1 was excluded because it used alternate-day fasting rather than daily TRE. The four supplementary reports [29,30,31,32] and the reason for each are listed in Table S2.

**Figure 1:**
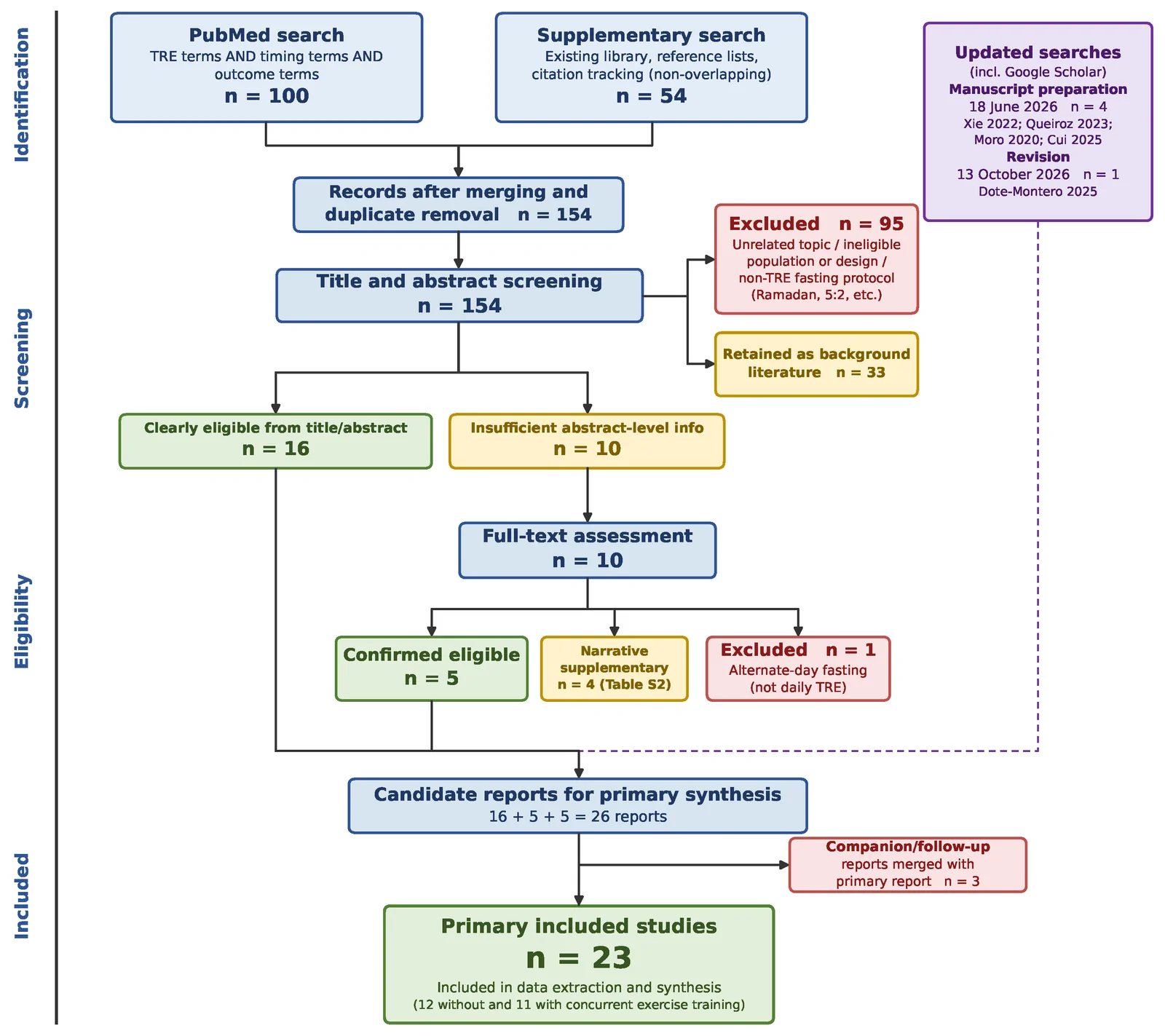
Literature search and screening flow diagram, adapted from the PRISMA framework for a narrative review [23,24,25,33,34]. The dashed branch indicates studies identified through updated searches during manuscript preparation and revision, which were not part of the initial 154 candidate records. The three merged companion or follow-up reports are identified in Section 2.4.

The updated searches identified 5 additional eligible studies: 4 during manuscript preparation [23,24,33,34] and 1 during revision [25], which had been overlooked in the earlier screening. This yielded 26 candidate reports (16 + 5 + 5). Three of these were companion or follow-up publications of an included trial and were merged with their primary report: a secondary-outcome report [35] of the trial by Miladi et al. [36] (same trial registration, PACTR202301473691345); the 6-month follow-up [37] of the trial by Črešnovar et al. [22] (NCT05730231); and the report by Richardson et al. [38], which analyzed the same 15 runners as Tovar et al. [39]. This left 23 independent studies for primary data extraction and synthesis. The selection process is summarized in Figure 1.

### 2.5. Data Extraction and Evidence Classification

Data were extracted from the 23 primary studies using a standardized framework covering study design and allocation; numbers of participants randomized, completing the intervention, and analyzed in each condition; participant characteristics (sex, age, weight status, physical activity, and training status); eating-window timing and duration; intervention duration; concurrent exercise training and its timing relative to the eating window; how energy intake was prescribed, assessed, or controlled, and whether intake was compared between conditions; total protein intake and, where reported, its distribution across meals; adherence to the eating window; body weight; body-composition method and outcomes; exercise-performance or physiological outcomes; and methodological or reporting issues relevant to interpretation. For between-condition comparisons, the reported mean difference and 95% confidence interval (CI) were extracted when available; otherwise, the reported test statistic or *p*-value was recorded. Study characteristics are summarized in Table 1 and Table 2. Within-group changes were distinguished from statistically supported between-group differences, and intention-to-treat and per-protocol results were distinguished when both were reported. Numerical inconsistencies, mathematically implausible values, incomplete reporting, and discrepancies between text and tables were recorded and considered in the methodological appraisal (Section 2.6).

The primary studies were grouped into three evidence categories. Direct timing-comparison studies compared early with midday, delayed, or late TRE under otherwise similar conditions. Indirect timing studies compared a single early or later TRE schedule with a non-TRE or energy-restriction control. Contextual studies used self-selected or individualized eating windows that could not be classified reliably as early or late. Only direct timing-comparison studies were used to evaluate the relative effects of earlier and later eating windows; indirect and contextual studies were used to characterize each type of schedule relative to non-TRE conditions and its compatibility with exercise training. The 4 studies retained as narrative supplementary evidence were not included in the primary synthesis and were cited only where they aided interpretation.

Exercise-performance outcomes were grouped into strength and muscular endurance, aerobic endurance and capacity, and anaerobic power or explosive performance, and, for all outcomes, studies with and without concurrent structured exercise were considered separately.

### 2.6. Methodological Appraisal

Each primary study was appraised at the study level across six domains: (1) allocation (randomized parallel, randomized crossover with or without counterbalanced order, or non-randomized); (2) attrition, overall and by condition; (3) analysis population (intention-to-treat or completers only); (4) energy intake, distinguishing an energy target that was only prescribed, intake that was assessed (and by which method), and intake that was controlled—for example, by providing meals; (5) assessment of adherence to the eating window; and (6) consistency of reported data across text, tables, and figures. Each study was then assigned a confidence level for its between-condition findings. Confidence was rated lower when allocation to the conditions being compared was non-randomized, when attrition exceeded 20% and only completers or a per-protocol sample were analyzed, when attrition differed by more than 10 percentage points between conditions, or when key outcome data were internally inconsistent; moderate when attrition exceeded 20% but all randomized participants were analyzed with likelihood-based or imputation methods, or when another domain was limited, such as completers-only analysis with low attrition or energy intake that was prescribed but not assessed; and higher otherwise. The appraisal was performed by one author (Z.Y.) and is reported in Table S3; the resulting confidence level is also shown for each study in Table 1 and Table 2. It was used to qualify the conclusions drawn from individual studies rather than to exclude studies from the synthesis.

### 2.7. Narrative Synthesis

Findings were synthesized narratively, and no pooled effect sizes were estimated, because the included studies differed substantially in participant characteristics, eating-window timing and duration, energy prescription, protein intake, intervention length, exercise protocol, and outcome assessment. Body composition was measured using methods including dual-energy X-ray absorptiometry, magnetic resonance imaging, bioelectrical impedance analysis, anthropometry, and ultrasonography, and exercise performance was assessed using heterogeneous tests unsuitable for quantitative pooling. The synthesis, therefore, focused on the direction and consistency of between-group findings within each evidence category; within-group changes were reported when informative but were not interpreted as evidence of superiority without a supporting between-group or group-by-time comparison.

Three further rules guided interpretation. First, a non-significant between-condition difference was treated as inconclusive rather than as evidence that an outcome was preserved or unaffected by eating-window timing; where CIs were available, they were reported to indicate the range of effects compatible with the data. Second, equivalent energy intake between conditions was assumed only when intake was controlled, for example by providing meals; a matched energy prescription, or a non-significant difference in self-reported intake, was not treated as evidence of equivalent intake. Third, patterns that emerged only from comparisons across studies, such as possible moderation of timing effects by concurrent exercise training or by energy intake, were treated as hypotheses, because no included study tested these interactions directly.

## 3. Results

### 3.1. Study Characteristics

Twenty-three independent intervention studies were included. Their characteristics and main findings are summarized in Table 1 (studies without concurrent structured exercise; n=12) and Table 2 (studies with prescribed or habitual structured training; n=11). Designs included parallel-group randomized, randomized crossover, and non-randomized controlled trials, with intervention durations ranging from 4 to 39 weeks; one trial also reported a 6-month follow-up after the intervention ended [37]. Study populations included healthy young adults, adults with overweight or obesity, older adults, physically inactive individuals, and resistance- or endurance-trained participants. Analyzed samples ranged from 15 to 197 participants, and 13 studies analyzed fewer than 50 participants.

Eight studies directly compared earlier and later eating windows (six without and two with concurrent training). In one of these, the later comparator was a midday window [23]; in another, allocation to the early or late window depended on chronotype [22]; and in a third, participants chose their own window [40]. Twelve studies compared a single early or later schedule with a non-TRE or energy-restriction control, including one trial in which windows of different lengths shared the same midpoint [41]. The remaining three used self-selected or individualized windows and were treated as contextual evidence [34,39,42].

Overall confidence in the between-condition findings was rated higher for one study [25], moderate for eight, and lower for fourteen (Section 2.6; Table S3). The most common reasons for a lower rating were attrition above 20% with only completer or per-protocol analysis, a between-condition difference in attrition of more than 10 percentage points, non-randomized allocation of the timing contrast, and internally inconsistent outcome data. Of the eight direct comparisons, one was rated higher, three moderate, and four lower in confidence.

### 3.2. Energy Intake, Adherence, and Exercise Timing

Ten studies prescribed an energy target or deficit (in one, the prescription was described inconsistently [36]), and 13 allowed ad libitum intake (Table 1 and Table 2). Achieved energy intake during the intervention was quantified in 14 studies, mostly by 3- to 7-day food or photographic records or by 24-h recalls. It was not quantified, or could not be estimated reliably, in the other nine [33,34,36,40,42,43,44,45,46]. No study provided all meals, and none tested whether intake was equivalent between conditions.

A shared energy prescription, therefore, did not ensure equal intake. In Queiroz et al., all three groups were prescribed the same 25% energy deficit, but reported intake reached only 59–67% of the prescribed amount [24]. In Tinsley et al. (2019), intake also remained below the prescribed target [47]. Likewise, a non-significant difference in measured intake did not establish equivalence. In Xie et al., energy intake decreased by 240 and 159 kcal/day with eTRF and mTRF, respectively (p=0.30) [23]. In the largest direct comparison, the early-minus-late difference was −143 kcal/day (95% CI, −403 to 118) [25]. Where intake did differ, it was higher during dTRE than during eTRE in our crossover trial (+64 kcal/day) [21]. In Jamshed et al., modeled intake was 214 kcal/day lower with eTRE than in the control group (95% CI, 12 to 416 kcal/day lower) [48].

Adherence to the eating window was assessed by self-report, meal photographs, or smartphone applications. Where it was quantified, adherence ranged from approximately 73% to 98% of participants or days, although definitions differed between studies. Five studies excluded non-adherent participants from their primary analysis [22,40,42,43,49], and two did so in a per-protocol analysis [41,47]. This practice can preferentially retain participants who tolerated the protocol well. Daily protein intake was reported in some studies, but no study reported how much protein was actually consumed at each meal; one study specified per-meal protein targets in its menus without verifying consumption [33].

In studies with structured training, exercise timing relative to the eating window varied. In four studies, training was scheduled at or near the end of the eating window or after it had closed, so participants could not eat after training until the next day’s window opened [34,36,46,50]. In Miladi et al., this also meant that the early and late groups trained at different clock times (16:00 vs. 20:00) [36]. In our 8-week trial, training took place at 19:00–20:00, after both the eTRE (08:00–14:00) and dTRE (12:00–18:00) windows had closed, whereas the control group could eat after training [46]. Training took place within the eating window or in the fed state in four studies [33,47,51,52]. One study manipulated fasted or fed training by design [42], and one applied TRE only on non-training days [49]. In the remaining study, exercise timing followed participants’ habitual training [39].

### 3.3. Body Composition

#### 3.3.1. Studies Without Concurrent Structured Exercise Training

Six studies without concurrent structured exercise directly compared earlier and later eating windows. None detected an advantage for the later window, and their findings for the earlier window differed by study design.

Three randomized trials did not prescribe an energy target. In the largest of these, and the only study rated higher in confidence, Dote-Montero et al. randomized 197 adults with overweight or obesity for 12 weeks [25]. The four groups were early 8-h TRE (starting by 10:00), late 8-h TRE (starting at or after 13:00), self-selected 8-h TRE, and usual care, and all groups received Mediterranean-diet education. All TRE groups lost more weight than usual care, but early and late TRE did not differ in body weight (−0.5 kg; 95% CI, −2.3 to 1.3), DXA-assessed fat mass (0.2 kg; −1.4 to 1.8), fat-free mass (−0.6 kg; −2.0 to 0.9), or MRI-assessed visceral adipose tissue (1%; −6 to 9). In Xie et al. (moderate confidence), eTRF reduced body weight relative to habitual eating (−1.6±1.4 vs. +0.3±1.2 kg; p=0.009), whereas mTRF did not (−0.2±2.2 kg) [23]. However, the direct eTRF–mTRF differences in body weight, fat mass, and body fat percentage (by BIA) were not statistically significant. In our crossover trial in 28 untrained young adults (moderate confidence), body weight decreased more during eTRE (08:00–14:00) than during dTRE (12:00–18:00) (difference, −0.95 kg; 95% CI, −1.74 to −0.16) [21]. Body composition was not measured in that trial, and energy intake was higher during dTRE.

Three studies prescribed an energy deficit. Queiroz et al. prescribed the same 25% deficit to eTRE (08:00–16:00), dTRE (12:00–20:00), and caloric restriction alone [24]. The three groups showed similar reductions in body weight (−4.2, −4.8, and −4.0 kg; group × time p=0.679), fat mass (−2.9, −3.6, and −3.1 kg; p=0.641), and fat-free mass (−1.2, −1.2, and −1.0 kg; p=0.828), all assessed by DXA. Confidence in this exploratory trial was lower because 23% of participants withdrew, attrition differed between arms by 12.5 percentage points, and the main analysis was per protocol; in addition, the prescribed deficit was not achieved. Crešnovar et al. found comparable weight loss with eTRE plus energy restriction, lTRE plus energy restriction, and energy restriction alone (−5.0, −4.4, and −4.3 kg; p=0.319) [22]. However, body fat percentage (by BIA) decreased more with eTRE than with lTRE (−1.2 percentage points; 95% CI, −2.1 to −0.2) and than with energy restriction alone (−1.1 percentage points; 95% CI, −2.0 to −0.1). Confidence in this comparison was lower. Participants with a morning chronotype could be allocated only to eTRE or energy restriction, and the others only to lTRE or energy restriction, so the eTRE–lTRE contrast also compared chronotypes. In addition, 24% of the lTRE group but only 8% of the eTRE group were lost before the per-protocol analysis. At the 6-month follow-up without dietary guidance, only 8–18% of participants still adhered on at least 5 days per week, and the earlier improvements were partly reversed [37]. Finally, in a non-randomized trial in which women chose both the diet and the window, weight loss was greater with an early than a late window (−10.06 vs. −4.32 kg; p=0.001), as was fat-mass loss (−6.47 vs. −3.45 kg) [40]. Several values in the early-versus-late table of that report duplicate other table entries, so these magnitudes are uncertain and cannot be separated from self-selection.

Taken together, none of the six direct comparisons found an advantage for the later window. Three reported an advantage for the earlier window. One of these was a small crossover trial in which body composition was not measured and intake was lower during eTRE; in the other two, the early-versus-late contrast was not randomized. The three randomized parallel-group trials, including the largest, did not detect a difference. In the largest trial, the confidence interval for body weight was compatible with differences of up to about 2 kg favoring the earlier window or about 1 kg favoring the later window. These findings are, therefore, inconclusive rather than evidence that eating-window timing has no effect. No trial varied both energy prescription and window timing, and the largest trial without an energy target did not detect a timing difference. The possibility that an eTRE advantage depends on whether energy intake is prescribed is, therefore, treated as a hypothesis in the Discussion.

Six studies compared a single TRE schedule with a non-TRE or energy-restriction control. Jamshed et al. (moderate confidence) reported greater weight loss with eTRE (07:00–15:00) plus energy restriction than with energy restriction over a window of at least 12 h (adjusted difference, −2.3 kg; 95% CI, −3.7 to −0.9) [48]. The fat-mass difference was not significant in the intention-to-treat analysis (−1.4 kg; 95% CI, −2.9 to 0.2), and fat-free mass and appendicular lean mass did not differ significantly. Because modeled intake was lower with eTRE, part of the weight difference may reflect energy intake. Thomas et al. (moderate confidence) added a 10-h window starting within 3 h of waking to daily caloric restriction [53]. This did not produce significant differences in weight, fat mass, or lean mass at 12 or 39 weeks. The median last meal in that trial was at 18:43 at week 12, so this window was early relative to waking rather than to clock time. In a crossover trial with lower confidence because 39% of randomized participants were not analyzed, four weeks of ad libitum eTRE (07:00–15:00) did not produce significant differences from habitual eating in body weight or BIA-assessed body composition [43].

Among trials of later or midday windows, the TREAT trial (moderate confidence for weight) found no significant difference in weight loss between lTRE (12:00–20:00) and consistent meal timing (−0.26 kg; 95% CI, −1.30 to 0.78) [44]. Appendicular lean mass index decreased more with lTRE (−0.16 kg/m^2^; 95% CI, −0.27 to −0.05). This lean-mass result came from a self-selected local subgroup of 50 participants and was one of many secondary outcomes, so confidence in it is lower. In men aged 65–74 years with overweight, an approximately 12:00–20:00 window was reported to reduce body weight by 1.92 kg, waist circumference by 3.11 cm, and visceral fat by 0.64 L relative to habitual eating [45]. However, the reported confidence intervals have signs that are inconsistent with the estimates, and the text and table disagree on the fat-free-mass result, so these magnitudes and the lean-mass finding are uncertain (lower confidence). Sampieri et al. (moderate confidence) compared 16:8 (10:00–18:00), 14:10, and 12:12 windows sharing a 14:00 midpoint with a normal diet [41]. Significant group × time interactions were found for body mass and fat mass: the 16:8 arm reduced body mass by 2.46% and fat mass by 8.65% without a significant change in lean soft tissue (DXA), whereas the other arms showed no significant changes. Because window length rather than clock time varied, this trial informs the length of the eating window rather than its timing.

These studies show that both earlier and later schedules reduced body weight relative to a control in some trials but not in others. Because the comparator was not an alternative TRE window, they do not indicate which timing is preferable. Lean-mass findings were inconclusive. Among these single-window versus non-TRE comparisons, the only significant lean-mass decrease came from a self-selected subgroup [44]. In the other studies, non-significant differences, mostly from small samples or BIA, do not demonstrate that lean mass was preserved.

#### 3.3.2. Studies with Concurrent Structured Exercise Training

Two trials directly compared earlier and later eating windows during matched training. Miladi et al. analyzed 61 women with overweight or obesity assigned to eTRE (08:00–16:00) or lTRE (12:00–20:00) combined with identical aerobic and resistance training three times per week (ETRE-PA and LTRE-PA), lTRE without exercise, or control [36]. Both exercise groups lost more weight than the lTRE-only and control groups (p<0.0005); no difference between ETRE-PA and LTRE-PA was reported. Within ETRE-PA, fat mass decreased by 7.44% and lean mass by 7.32%, but neither change was supported by a significant group × time interaction. Confidence was lower because 25% of each exercise group was lost before the completer analysis, participant flow was reported inconsistently, and intake was not assessed. In our 8-week trial in 24 young women performing standardized push-up training (moderate confidence), body weight changed by −2.61±1.06 kg with eTRE (08:00–14:00), −1.44±1.12 kg with dTRE (12:00–18:00), and −0.48±0.64 kg in the control group [46]. Triceps brachii thickness increased by 1.36–1.55 mm in all groups, with no significant interaction with time for eTRE (p=0.780) or dTRE (p=0.506) relative to control. A direct eTRE–dTRE estimate was not reported, and whole-body composition was not measured. With 8 to 15 participants per arm, neither trial can rule out moderate timing differences.

Six trials compared TRE plus a training program with the same program under normal eating. Fat mass decreased more with TRE in four of them. These were Ameur et al., using eTRE (08:00–16:00) with high-intensity functional training (greater fat-mass reduction than with eTRE alone, p<0.01, or training alone, p<0.001) [50], and Kotarsky et al., using lTRE (12:00–20:00) with concurrent training (approximately −3 vs. −1 kg; group × time p=0.002) [52]. They also included Moro et al. (2016), using an approximately 13:00–21:00 window with resistance training (−1.61 vs. −0.30 kg; ANCOVA p=0.007) [51]. The fourth was Cui et al., using a self-selected 10-h window (mean 08:14–17:50) with resistance training, which reduced fat mass by 3.2 kg, compared with 1.1 kg for resistance training alone and 0.3 kg for TRE alone (BIA) [34]. No significant effect on fat mass was detected with a 4-h window applied only on non-training days [49]. In resistance-trained women following lTRE, a fat-mass advantage appeared only in the per-protocol analysis [47]. Moro et al. (2016) [51] was rated moderate in confidence and the other five trials lower. Because the windows were early, later, or self-selected and no trial compared timings within the same study, these results indicate that additional fat loss with TRE during training was observed with each type of window, but not whether its size depends on timing.

None of the five trials with interpretable lean-mass data detected a significant difference in lean or fat-free mass between TRE plus training and training alone. These null results included Kotarsky et al. (approximately 0 vs. +1 kg; group × time p=0.292) [52], Moro et al. (2016) [51], and the intention-to-treat analysis of Tinsley et al. (2019) [47], as well as lean soft tissue in Tinsley et al. (2017) [49]. In Cui et al., fat-free mass decreased by 2.3 kg with TRE alone, changed by +0.2 kg with TRE plus resistance training, and increased by 1.6 kg with resistance training alone (BIA; intake not recorded) [34]. Fat-free-mass values in Ameur et al. were internally inconsistent and could not be interpreted [50]. Between-condition confidence intervals for lean mass were not reported in these trials, and arms included 8 to 17 analyzed participants. These null results, therefore, do not establish that lean mass is maintained during TRE with training. The within-group lean-mass decrease with ETRE-PA in Miladi et al. shows that training did not prevent lean-mass loss in every study [36].

In trained participants who maintained their habitual endurance training, TRE was accompanied by modest reductions in body mass or fat mass. Among 16 elite male cyclists, an approximately 10:00–18:00 window reduced body weight relative to habitual eating (−1.26 vs. +0.22 kg; group × time p=0.04), with no significant change in fat-free mass [33]. Confidence was lower because baseline fat-mass values were reported inconsistently. In 15 trained male runners, a self-selected 8-h window reduced DXA-assessed fat mass by approximately 0.8 kg and body fat percentage by approximately 1 percentage point, with no significant change in lean mass [38,39]. A sequence effect was present for body fat percentage, and these two publications report the same cohort. Hofstätter et al. found that body-weight change differed between individualized 16:8 TRE groups training in the fed or fasted state and an ad libitum control (p=0.002) [42]. Its body-fat results could not be interpreted because the published table reports negative standard deviations.

Across the exercise studies, additional fat loss relative to training alone was reported in four of six trials using early, later, or self-selected windows, and neither direct comparison detected an early-versus-late difference. Two related ideas are discussed as hypotheses rather than findings: that structured training reduces the influence of window timing, and that resistance training protects lean mass during TRE. This is because both rest on comparisons across different studies, such as the lean-mass decrease with lTRE alone in the TREAT trial [44] versus the absence of detected lean-mass loss with lTRE plus resistance training [47,51,52], rather than on tested interactions.

### 3.4. Exercise Performance

Performance data came from three situations. The first was a new training program in previously untrained or recreationally active participants, which tests adaptation to training. The second was continuation of habitual training in athletes [33,39], which tests whether existing performance is maintained. The third was no structured training [21,24]. These situations are distinguished below where relevant.

#### 3.4.1. Strength and Muscular Endurance

Neither of the two direct comparisons detected a timing-related difference in strength or muscular endurance. In Miladi et al., leg-extension one-repetition maximum (1RM), 30-s squat, and 30-s crunch performance improved more in both ETRE-PA and LTRE-PA than in the non-exercise groups, and bench-press 1RM improved within both exercise groups [36]. No difference between ETRE-PA and LTRE-PA was reported, and no between-group estimate was given. Because only these two groups trained, their advantage over the non-exercise groups reflects the training stimulus rather than an effect of TRE. In our trial, push-up repetitions to failure increased by approximately 62–74 repetitions in the eTRE, dTRE, and control groups, with no significant eTRE-by-time or dTRE-by-time interaction (both p=0.486) [46]. The reported interaction tests compared each TRE group with the control group; a specific eTRE–dTRE contrast in changes in push-up performance was not reported, so these results do not establish whether the two windows differed.

Among trials comparing a single TRE schedule with exercise-matched normal eating, changes in bench-press 1RM (+3.3 vs. +0.8 kg) and leg-press 1RM (+7.2 vs. +10.4 kg) did not differ significantly between lTRE and a normal diet in resistance-trained men [51]. In Kotarsky et al., knee-extension peak torque showed a significant group × time interaction, increasing by 13.5% with normal eating and decreasing by 4.8% with lTRE [52]. Post hoc comparisons were not significant, and other strength and endurance measures did not differ. Group × time interactions for strength and muscular endurance were non-significant in untrained men using a 4-h window on non-training days (p=0.07–0.97) [49] and largely non-significant in resistance-trained women following lTRE [47].

Strength and muscular endurance, thus, improved in most groups that trained, whatever the eating window. However, no study reported a between-condition estimate with a confidence interval for these outcomes, arms included 8 to 17 analyzed participants, and one trial detected an interaction favoring normal eating for knee-extensor torque [52]. The evidence, therefore, does not show that TRE or its timing alters strength adaptation, but it is also insufficient to show that adaptation is unaffected.

#### 3.4.2. Aerobic Endurance and Aerobic Capacity

The aerobic outcomes measured different aspects of performance: time to exhaustion at a fixed submaximal workload, peak or maximal oxygen uptake in the laboratory, functional walking capacity (6-min walk test), and competitive endurance performance (a 10-km time trial).

Three trials directly compared earlier and later windows on aerobic outcomes in non-athletes, and none detected a timing-related difference. In our crossover trial without exercise, cycling time to exhaustion at a fixed workload was 8.22 min at baseline, 8.23 min after eTRE, and 8.33 min after dTRE (all pairwise effect sizes $d_{z}<0.2$; no confidence interval reported) [21]. Under prescribed caloric restriction, peak oxygen uptake changed by +0.1 (95% CI, −2.6 to 2.9), −0.3 (−2.0 to 1.3), and −0.8 (−2.8 to 1.0) mL/kg/min with eTRE, dTRE, and caloric restriction alone (group × time p=0.817) [24]. With concurrent training, 6-min walk distance improved more in both ETRE-PA and LTRE-PA than in the control group (group × time p<0.0005; $\eta_{p}^{2}=0.55$), but no ETRE-PA versus LTRE-PA estimate was reported [36].

In trained athletes maintaining their habitual training, the two available studies did not compare early and later windows. Among 16 elite cyclists, absolute maximal oxygen uptake changed from 4.83 to 4.59 L/min with an approximately 10:00–18:00 window and from 4.90 to 4.85 L/min with habitual eating, with no significant group × time interaction [33]. Absolute peak power output in the incremental test also showed no significant interaction (407.5 to 415.0 W vs. 411.3 to 403.8 W). Peak power output relative to body mass showed a significant interaction (6.08 to 6.31 vs. 5.71 to 5.60 W/kg; p=0.024), although the within-group increase with TRE was not significant (p=0.06). With eight cyclists per group, the numerically larger decrease in absolute maximal oxygen uptake with TRE can be neither confirmed nor excluded. In 15 trained male runners, a self-selected 8-h window changed 10-km treadmill time-trial performance by −0:20±3:34 min:s, compared with −0:36±2:57 min:s with habitual eating, from baseline times of approximately 49–50 min (p=0.53; no confidence interval reported) [39]. The mean difference in change (about 16 s, approximately 0.5% of finishing time) was much smaller than the standard deviations of change (about 3–3.5 min). The trial, therefore, cannot exclude differences of a size relevant to competitive performance. The time trial was run fasted after a graded substrate-utilization test.

At the physiological level, fat-oxidation capacity improved over time with individualized 16:8 TRE combined with aerobic training [42]. However, group × time interactions were not significant for fat oxidation at a matched workload (p=0.371) or for maximal fat oxidation (p=0.262).

No included study detected an early-versus-late difference in an aerobic outcome, but the three direct comparisons assessed submaximal endurance, peak oxygen uptake, and walking capacity in non-athletes. None assessed trained athletes or competitive performance. In athletes, the available data come from two small studies without an early-versus-late contrast. In cyclists, the change in relative peak power coincided with body-mass loss rather than higher absolute power [33]. These findings indicate that no statistically significant decrements were detected over 4 to 12 weeks, but they do not establish that aerobic capacity or endurance performance is maintained during TRE, or that it is unaffected by window timing.

#### 3.4.3. Anaerobic Power and Explosive Performance

Anaerobic power was the only outcome for which a later eating window was associated with a better result than an earlier one. In our crossover trial in 28 untrained young adults without concurrent structured exercise (moderate confidence), the highest peak power output across three 10-s sprints was 581.8 W at baseline, 603.0 W after eTRE, and 617.2 W after dTRE [21]. The dTRE–eTRE difference was 14.18 W (95% CI, 1.79 to 26.57; $d_{z}=0.54$), approximately 2% of the post-eTRE value. In the same participants, body weight decreased more during eTRE.

Three features of the trial limit what this difference can establish. First, all tests were performed between 15:00 and 17:00, which fell at or shortly after the close of the eTRE window (last meal 14:00–15:00) but in the middle of the dTRE window, and pre-test intake was not reported. The difference may, therefore, reflect nutritional state on the test day rather than an adaptation. Second, peak power increased after both conditions although participants did not train, which is consistent with continued learning despite one familiarization session. Third, sequences were allocated by simple randomization and the number of participants in each sequence was not reported, no new baseline was measured after the washout, and the analysis did not include period or sequence terms. If learning continued across test sessions, the condition tested last would be favored, and any imbalance between sequences could bias the dTRE–eTRE contrast. The trial, therefore, shows a short-term difference in afternoon sprint power under the two eating schedules, but not whether this reflects a lasting adaptation to dTRE.

When TRE was combined with exercise training, no timing-related difference in explosive performance was reported. In Miladi et al., vertical-jump height improved more in both ETRE-PA and LTRE-PA than in the non-exercise groups, without a reported difference between the two exercise groups [36]. In resistance-trained women, countermovement-jump performance, peak force, and rate of force development did not differ among lTRE, lTRE plus supplementation, and normal eating [47]. Peak power output in elite cyclists [33] was measured during an incremental test, reflects aerobic rather than anaerobic capacity, and is, therefore, considered in the preceding section.

**Table 1:** Characteristics and main findings of studies conducted without concurrent structured exercise training.

Study	Design and Participants	TRE Protocol and Comparator	Duration	Exercise Context	Body-Weight and Body-Composition Findings	Exercise-Performance or Physiological Findings	Confidence
Xie et al. (2022) [23]	Three-group RCT; 90 adults without obesity randomized, 82 completers analyzed (eTRF, n=28; mTRF, n=26; control, n=28).	eTRF: ≤8 h between 06:00 and 15:00; mTRF: ≤8 h between 11:00 and 20:00; ad libitum control.	5 weeks	No structured exercise prescribed.	Body-weight reduction was greater with eTRF than control (−1.6±1.4 vs. +0.3±1.2 kg; p=0.009). Fat mass and body-fat percentage also decreased more with eTRF than control (BIA). No significant body-composition differences were detected between eTRF and mTRF.	Exercise performance was not assessed.	Moderate
Yu and Ueda (2025) [21]	Randomized crossover trial; 28 healthy, untrained university students (16 women, 12 men), all completed both conditions; no new baseline after the washout.	eTRE: 08:00–14:00; dTRE: 12:00–18:00; two intervention periods separated by a 2-week washout.	4+4 weeks	No structured exercise prescribed.	Body weight decreased during both conditions, more during eTRE than dTRE (difference −0.95 kg, 95% CI −1.74 to −0.16; p=0.020). Energy intake was higher during dTRE (+64 kcal/day; p=0.040). Formal body composition was not assessed.	Cycling time to exhaustion did not differ between conditions. Peak power increased during both conditions, more during dTRE (difference +14.18 W, 95% CI 1.79 to 26.57; p=0.025); tests at 15:00–17:00 fell after the eTRE window but within the dTRE window.	Moderate
Crešnovar et al. (2025) [22]	Three-group parallel trial; 108 adults with overweight or obesity allocated, 93 completers analyzed per protocol. Randomization to TRE or ER occurred within chronotype strata, so eTRE versus lTRE was not randomized.	eTRE+ER: 08:00–16:00; lTRE+ER: 12:00–20:00; ER-only control. All groups were prescribed an approximately 500-kcal/day deficit.	3 months	No structured exercise prescribed.	Weight loss was comparable across eTRE+ER, lTRE+ER, and ER (−5.0, −4.4, and −4.3 kg; p=0.319). Body-fat percentage decreased more with eTRE+ER than with lTRE+ER (difference −1.2 percentage points, 95% CI −2.1 to −0.2; p=0.013) and ER alone (p=0.022) (BIA).	Exercise performance was not assessed.	Lower (allocation by chronotype)
Queiroz et al. (2023) [24]	Three-group RCT; 48 adults aged 20–40 years with overweight or obesity randomized, 37 completed (13/11/13); per-protocol results reported, ITT in the supplement.	eTRE+CR: 08:00–16:00; dTRE+CR: 12:00–20:00; CR control: 08:00–20:00. All groups were prescribed a 25% energy deficit; reported intake was 59–67% of the prescription.	8 weeks	Usual physical activity maintained; no structured training.	All groups lost body weight, fat mass, and fat-free mass, with no significant group-by-time interactions (DXA). Weight loss was −4.2, −4.8, and −4.0 kg in eTRE, dTRE, and CR, respectively.	Peak oxygen uptake changed by +0.1 (95% CI −2.6 to 2.9), −0.3 (−2.0 to 1.3), and −0.8 (−2.8 to 1.0) mL/kg/min in eTRE, dTRE, and CR; group-by-time p=0.817.	Lower (attrition)
Dote-Montero et al. (2025) [25]	Four-group multicenter RCT; 197 adults aged 30–60 years with overweight or obesity randomized (UC 49, early 49, late 52, self-selected 47); all 197 analyzed (ITT).	8-h windows: early TRE starting by 10:00 (median 09:45–17:30); late TRE starting at or after 13:00 (median 14:20–21:30); self-selected TRE. All arms, including the UC control, received Mediterranean-diet education; no energy target.	12 weeks	No structured exercise prescribed; accelerometer-measured activity did not change.	All TRE arms lost more weight than UC (early, −2.9 kg, 95% CI −4.7 to −1.1; late, −2.4 kg, −4.2 to −0.6). Early versus late TRE: weight −0.5 kg (−2.3 to 1.3), fat mass 0.2 kg (−1.4 to 1.8), fat-free mass −0.6 kg (−2.0 to 0.9) (DXA), and visceral adipose tissue 1% (−6 to 9) (MRI); none significant.	Exercise performance was not assessed.	Higher
Dhieb et al. (2025) [40]	Non-randomized controlled trial; 100 women with obesity chose IF (45) or CCR (55); 75 completed. Within IF, participants chose an early or a late window (16 and 14 completers).	IF: 8-h window 08:00–16:00 (early) or 12:00–20:00 (late), two meals and a snack; CCR control. Both were prescribed a 25% energy deficit.	3 months	General advice to walk 30–60 min daily; no structured training.	Within IF, weight loss was larger with the early than the late window (−10.06 vs. −4.32 kg; p=0.001), as was fat-mass loss (−6.47 vs. −3.45 kg). Several early-window values in the source table duplicate other table entries, so these magnitudes are uncertain.	Exercise performance was not assessed.	Lower (non-randomized; data errors)
Jamshed et al. (2022) [48]	Parallel RCT; 90 adults with obesity randomized and analyzed (ITT); 59 completed all testing.	eTRE+ER: 07:00–15:00; control eating schedule of ≥12 h combined with ER.	14 weeks	No structured exercise prescribed.	eTRE+ER produced greater weight loss than control (−2.3 kg, 95% CI −3.7 to −0.9; p=0.002). The fat-mass difference was not significant in the ITT analysis (−1.4 kg, 95% CI −2.9 to 0.2; p=0.09); fat-free mass and appendicular lean mass did not differ significantly (DXA).	Exercise performance was not assessed.	Moderate
Thomas et al. (2022) [53]	Parallel RCT; 85 adults with obesity randomized, 81 started (69 women); 70 retained at week 12 and 63 at week 39.	10-h E-TRE starting within 3 h of waking (median last meal 18:43 at week 12) plus daily caloric restriction versus daily caloric restriction alone.	39 weeks; primary assessment at week 12	No structured exercise prescribed.	Weight loss and DXA-derived changes in fat mass and lean mass did not differ significantly between groups at 12 weeks (DXA in a subset) or 39 weeks.	Exercise performance was not assessed.	Moderate
Mena-Hernández et al. (2024) [43]	Randomized crossover trial; 28 randomized, 17 adults with overweight or obesity analyzed (12 women, 5 men) after withdrawals and adherence exclusions.	eTRE: 07:00–15:00; habitual ad libitum eating condition; 4-week washout.	4+4 weeks	No structured exercise prescribed.	No significant between-condition differences were observed in body weight, fat mass, body-fat percentage, or fat-free mass (BIA).	Exercise performance was not assessed.	Lower (attrition)
Lowe et al. (2020), TREAT trial [44]	Parallel RCT; 141 adults with overweight or obesity randomized, 116 with data analyzed, 105 completed; DXA in a self-selected local subgroup (n=50).	lTRE: 12:00–20:00; consistent-meal-timing control.	12 weeks	No structured exercise prescribed.	Weight loss did not differ significantly between groups (−0.26 kg, 95% CI −1.30 to 0.78; p=0.63). In the DXA subgroup, appendicular lean mass index decreased more with lTRE than control (−0.16 kg/m^2^, 95% CI −0.27 to −0.05; p=0.005).	Exercise performance was not assessed.	Moderate (lean mass: lower)
Domaszewski et al. (2022) [45]	Two-group parallel trial reported as randomized; 46 men aged 65–74 years with overweight (n=23 per group).	lTRE: 16-h daily fast with an eating window of about 12:00–20:00 (clock times reported ambiguously); habitual-diet control.	6 weeks	No structured exercise prescribed.	Compared with control, lTRE reduced body weight by 1.92 kg, visceral fat by 0.64 L, and waist circumference by 3.11 cm. Skeletal muscle mass did not change significantly; reported CIs and the fat-free-mass result are internally inconsistent.	Exercise performance was not assessed.	Lower (inconsistent data)
Sampieri et al. (2024) [41]	Four-group RCT; 41 healthy, non-trained adults randomized, 40 analyzed (ITT) and 32 per protocol.	Fixed windows: 16:8, 10:00–18:00; 14:10, 09:00–19:00; 12:12, 08:00–20:00; ad libitum normal diet. Window length varied while the midpoint stayed at 14:00.	8 weeks	No structured exercise prescribed.	Significant group-by-time interactions for body mass and fat mass: in the 16:8 arm, body mass fell by 2.46% and fat mass by 8.65%, with no change in lean soft tissue (DXA); the 14:10 and 12:12 arms showed no significant changes.	Exercise performance was not assessed.	Moderate

Notes: Studies in this table did not include structured exercise training during the intervention, whether prescribed by the investigators or maintained as part of participants’ habitual routine; where reported, participants were instructed to maintain their usual physical activity. Studies are ordered as direct timing comparisons followed by indirect timing studies. Within-group changes are not interpreted as evidence that one timing strategy was superior unless supported by a statistically appropriate between-group or group-by-time comparison, and non-significant differences are not interpreted as equivalence. Confidence is the overall confidence in each study’s between-condition findings from the methodological appraisal (Section 2.6; Table S3), with the main reason for a lower rating in parentheses. BIA, bioelectrical impedance analysis; CCR, continuous caloric restriction; CI, confidence interval; CR, caloric restriction; DXA, dual-energy X-ray absorptiometry; dTRE, delayed time-restricted eating; ER, energy restriction; eTRE/eTRF, early time-restricted eating/feeding; IF, intermittent fasting; ITT, intention-to-treat; lTRE, late time-restricted eating; MRI, magnetic resonance imaging; mTRF, midday time-restricted feeding; RCT, randomized controlled trial; UC, usual care.

**Table 2:** Characteristics and main findings of studies combining time-restricted eating with structured exercise training.

Study	Design and Participants	TRE Protocol and Comparator	Duration	Exercise Intervention	Body-Weight and Body-Composition Findings	Exercise-Performance or Physiological Findings	Confidence
Miladi et al. (2025) [36]	Four-group RCT; 75 allocated, 61 sedentary women with overweight or obesity analyzed as completers (ETRE-PA 15, LTRE-PA 15, LTRE 15, control 16); participant flow reported inconsistently.	ETRE-PA: 08:00–16:00; LTRE-PA: 12:00–20:00; LTRE without exercise; non-intervention control.	12 weeks	Combined aerobic and resistance exercise three times per week, held at the end of each eating window (16:00 vs. 20:00).	Weight loss was greater in both exercise groups than in LTRE alone or control. Fat mass decreased significantly within ETRE-PA (−7.44%), but the group-by-time interaction was not significant. Lean mass also decreased within ETRE-PA (−7.32%) (BIA).	Both exercise groups improved multiple fitness tests, including the 6-min walk, strength-endurance tests, and vertical jump; no difference between ETRE-PA and LTRE-PA was reported.	Lower (attrition; flow)
Yu and Ueda (2025) [46]	Three-group RCT; 24 healthy young women with limited resistance-training experience (n=8 per group), all analyzed.	eTRE: 08:00–14:00; dTRE: 12:00–18:00; unrestricted-timing control.	8 weeks	Supervised knee-supported push-up training, 4×10 repetitions, three times per week at 19:00–20:00, after both TRE windows had closed.	Body weight decreased most with eTRE. Triceps-brachii muscle thickness increased by approximately 1.36–1.55 mm in all groups, with no significant between-group difference (ultrasound). Whole-body fat and lean mass were not measured.	Push-up repetitions to failure increased by approximately 62–74 repetitions in all groups, with no significant group-by-time interaction.	Moderate
Moro et al. (2020) [33]	Two-group RCT; 16 young elite male road cyclists (8 per group), all analyzed.	TRE: nominal 8-h window (approximately 10:00–18:00/19:00); control: habitual eating (approximately 07:00–21:00). Not a direct early-versus-later comparison.	4 weeks	Habitual pre-competition endurance training maintained (6 sessions/week; approximately 500 km/week); no new exercise prescribed.	Body weight decreased with TRE and increased slightly with control (−1.26 vs. +0.22 kg; group × time p=0.04). Fat-free mass did not change significantly, and group × time effects on fat-related outcomes were not significant (BIA); baseline fat-mass values are reported inconsistently.	Absolute peak power output and VO_2max_ showed no significant group × time interaction. Peak power output relative to body mass showed a significant interaction (p=0.024), although the within-group change with TRE was not significant (p=0.06). Peak power was derived from an incremental test rather than a sprint test.	Lower (inconsistent data)
Tovar et al. (2021) and Richardson et al. (2023) [38,39]	Randomized crossover trial; 19 randomized, 15 endurance-trained male middle- and long-distance runners completed both conditions (8 TRF-first). The two publications report the same cohort.	Self-selected 8-h TRE window versus a habitual approximately 12-h eating window; washout planned as 2 weeks (mean 2.1 weeks).	4+4 weeks	Habitual endurance training maintained; no new standardized training program prescribed.	TRE reduced whole-body fat mass by approximately 0.8 kg and body-fat percentage, with no significant change in lean mass (DXA); a sequence effect was present for body-fat percentage.	10-km treadmill time trial (run was faster after a graded test) changed by −0:36±2:57 (habitual) vs. −0:20±3:34 min:s (TRE); p=0.53; no CI reported.	Lower (attrition)
Cui et al. (2025) [34]	Four-group RCT (control, TRE, RT, TRE+RT); 58 physically active university students with overweight or obesity randomized, 54 completers analyzed (n=12, 15, 13, and 14).	Self-selected 10-h window within 08:00–20:00 (actual mean approximately 08:14–17:50); control and RT groups maintained habitual eating. Intake not recorded.	8 weeks	Supervised resistance training, 3 sessions/week at 60–70% 1RM, 16:00–18:00 (RT and TRE+RT groups).	Body weight decreased more with TRE (−2.6 kg) and TRE+RT (−2.9 kg) than with control. Fat-mass reduction was greatest with TRE+RT (−3.2 kg) compared with TRE (−0.3 kg) and RT (−1.1 kg) (BIA). Fat-free mass decreased with TRE alone (−2.3 kg), increased with RT (+1.6 kg), and did not change significantly with TRE+RT (+0.2 kg).	Exercise performance was not reported. No significant group × time interactions were observed for blood pressure, mood, or sleep.	Lower (attrition gap)
Ameur et al. (2024) [50]	Three-group RCT; 74 randomized, 64 physically inactive women with obesity analyzed as completers (TRE 20, HIFT 24, TRE-HIFT 20).	eTRE: 08:00–16:00; TRE alone, HIFT alone, and combined TRE-HIFT.	12 weeks	High-intensity functional training three times per week at 17:00, after the TRE window, in HIFT and TRE-HIFT.	All groups lost weight. Fat-mass reduction was greater with TRE-HIFT than with TRE alone (p<0.01) or HIFT alone (p<0.001). Reported fat-free-mass values are internally inconsistent and could not be interpreted reliably.	No separate exercise-performance outcome was reported.	Lower (inconsistent data)
Moro et al. (2016) [51]	Parallel RCT; 34 men with at least 5 years of resistance-training experience (n=17 per group), all analyzed.	lTRE: meals at 13:00, 16:00, and 20:00; normal-diet control (08:00, 13:00, 20:00); matched energy targets.	8 weeks	Supervised resistance training three times per week at approximately 85–90% of 1RM, 16:00–18:00.	Fat mass decreased more with lTRE than with the normal diet (−1.61 vs. −0.30 kg; ANCOVA p=0.007) (DXA). Fat-free mass did not change significantly in either group.	Leg-press 1RM increased over time; bench-press and leg-press changes did not differ between groups.	Moderate
Kotarsky et al. (2021) [52]	Parallel RCT; 23 previously inactive adults aged 35–60 years with overweight or obesity randomized, 21 completed (TRE 11, control 10).	lTRE: 12:00–20:00; normal-duration eating condition with the same exercise program.	8 weeks	Supervised resistance training and progressively increased aerobic training, performed in the fed state.	Body weight decreased by 3.3% with TRE versus 0.2% with control. Fat mass decreased by approximately 3 versus 1 kg (9.0% vs. 3.3%; group × time p=0.002). Whole-body lean mass changed by approximately 0 versus +1 kg (group × time p=0.292) (DXA).	Knee-extension peak torque showed a group × time interaction (+13.5% with normal eating vs. −4.8% with TRE), although post hoc comparisons were not significant; other strength and endurance measures did not differ between groups.	Lower (attrition gap)
Tinsley et al. (2017) [49]	Parallel RCT; 28 untrained young men started, 18 analyzed (TRF, n=10; normal diet, n=8) after withdrawals and adherence exclusions.	TRF: 4-h window self-selected within 16:00–24:00, applied on non-training days only; normal-diet control.	8 weeks	Whole-body resistance training three times per week.	No significant group-by-time interaction was observed for body weight or lean soft tissue.	Strength and muscular-endurance adaptations generally did not differ between groups.	Lower (attrition)
Tinsley et al. (2019) [47]	Three-group RCT; 40 resistance-trained young women randomized and analyzed (ITT); 31 completed and 24 met per-protocol criteria.	lTRE: 12:00–20:00 with HMB or placebo; normal-eating comparator.	8 weeks	Supervised resistance training three times per week in the fed state; protein intake approximately 1.6 g/kg/day.	Intention-to-treat analyses showed no significant between-group differences in fat-free mass or muscle thickness. A possible fat-mass advantage appeared only in the per-protocol analysis.	Strength and other performance outcomes improved across groups, with no consistent between-group differences.	Lower (attrition gap)
Hofstätter et al. (2025) [42]	Three-group RCT; 52 physically active adults entered, 36 analyzed after withdrawals and adherence exclusions.	Individualized 16:8 TRE combined with exercise in the fasted or fed state; unrestricted-eating exercise control. The windows were not fixed as early or late.	8 weeks	Fatmax-oriented aerobic training at 08:00–13:00 after a carbohydrate snack, in the fasted state, or without TRE.	Body-weight change differed among groups, with the largest reduction in the fed-before-training TRE group. Body-fat and muscle-percentage values could not be interpreted reliably because the published table contains implausible negative standard deviations.	Fatmax-related outcomes improved over time, but group-by-time interactions were not significant for matched-load or maximal fat oxidation.	Lower (attrition; data)

Notes: Studies in this table included structured exercise training during the intervention, either prescribed by the investigators or maintained as part of participants’ habitual training, as in the elite cyclists [33] and trained runners [38,39]. Tovar et al. and Richardson et al. reported complementary outcomes from the same cohort and were counted as one independent study. Within-group changes are not interpreted as evidence that one timing strategy was superior unless supported by a statistically appropriate between-group or group-by-time comparison, and non-significant differences are not interpreted as equivalence. Confidence is the overall confidence in each study’s between-condition findings from the methodological appraisal (Section 2.6; Table S3), with the main reason for a lower rating in parentheses. 1RM, one-repetition maximum; BIA, bioelectrical impedance analysis; CI, confidence interval; DXA, dual-energy X-ray absorptiometry; eTRE/ETRE, early time-restricted eating; dTRE, delayed time-restricted eating; Fatmax, exercise intensity eliciting maximal fat oxidation; HIFT, high-intensity functional training; HMB, β-hydroxy-β-methylbutyrate; ITT, intention-to-treat; lTRE/LTRE, late time-restricted eating; PA, physical activity; RCT, randomized controlled trial; RT, resistance training; TRF, time-restricted feeding; VO_2max_, maximal oxygen uptake.

Overall, the evidence does not establish an advantage of either timing condition for anaerobic power or explosive performance. The dTRE advantage in sprint power comes from a single trial with the limitations described above, and whether training modifies it cannot be judged, because the trials that included training used different tests, populations, and comparators.

## 4. Discussion

### 4.1. Principal Findings

This narrative review synthesized 23 intervention studies examining earlier and later TRE in relation to body composition and exercise performance. Four principal findings emerged.

First, none of the eight direct comparisons found a body-composition advantage for the later window, but the evidence for an advantage of the earlier window was inconclusive. Three comparisons favored the earlier window. One was a small crossover trial without body-composition measurement [21]; in the other two, the early-versus-late contrast was not randomized [22,40]. The randomized parallel-group trials, including the largest and only study rated higher in confidence, did not detect differences [23,24,25]. The current evidence, therefore, does not support a general body-composition advantage for eTRE, but it is also too imprecise to exclude a small one.

Second, when TRE was added to structured training, fat mass decreased more than with training alone in four of six trials, using early, later, or self-selected windows [34,50,51,52]. No trial with interpretable data detected a difference in lean mass, but these comparisons were small and did not report confidence intervals.

Third, no direct comparison detected an early-versus-late difference in strength, muscular endurance, or aerobic outcomes [21,24,36,46]. Because the estimates were imprecise, these findings indicate only that large differences were not detected; they do not demonstrate that performance was maintained or that it is unaffected by timing.

Fourth, the only outcome that favored the later window was sprint power in one crossover trial without training [21], and features of its design limit what this result can establish.

Taken together, the evidence answers the question in the title negatively: earlier was not consistently better. It does not show, however, that timing is irrelevant. Whether the effect of timing depends on energy intake, exercise, or the outcome assessed remains a hypothesis that the available studies were not designed to test.

### 4.2. Interpretation of Body-Composition Findings

#### 4.2.1. Energy Intake as a Candidate Moderator

This hypothesis arises because weight differences favoring the earlier window appeared in trials without an energy target [21,23] but not when all groups were prescribed the same deficit [24,53]. This pattern rests on comparisons across studies that differed in population, duration, window definitions, and measurement methods, rather than on a tested interaction between timing and energy prescription. It is also weakened by the largest trial in the review, which did not prescribe an energy target and found no early-versus-late difference in body weight, fat mass, or visceral fat [25]. Energy intake should, therefore, be regarded as a candidate moderator, not an established one.

Two features of the evidence make this question difficult to resolve. First, a prescribed deficit is not the same as achieved intake: in Queiroz et al., reported intake reached only 59–67% of the prescription [24], so the groups were matched by instruction rather than by consumption. Second, where intake was measured, it was mostly self-reported over a few days, and a non-significant difference between conditions does not establish that intake was equivalent. For this reason, the greater improvement in insulin resistance with eTRF than with mTRF in Xie et al. [23] cannot be attributed to timing independently of intake, because the reductions in energy intake (240 vs. 159 kcal/day; p=0.30) were not shown to be equal. Similarly, in our crossover trial the greater weight loss during eTRE was accompanied by lower intake [21], and in Jamshed et al. part of the weight advantage of eTRE may reflect a modeled intake that was 214 kcal/day lower [48].

The most direct evidence for an intake-independent effect of timing comes from a supplementary study in which all meals were provided and energy intake was matched [29]. In that crossover trial, body weight changed by −1.1 kg with daytime eating (08:00–19:00) and by +0.2 kg with delayed eating (12:00–23:00) over 8 weeks (p=0.074; n=12), and insulin resistance improved more with daytime eating. Because its 11-h windows exceed the TRE definition used here and the sample was small, this study is consistent with a modest effect of timing but cannot confirm it. Testing the hypothesis would require trials that cross window timing with controlled or verified energy intake.

#### 4.2.2. Methodological Quality and Durability

The two non-randomized contrasts that favored eTRE relied on BIA and could not separate timing from participant characteristics. In Crešnovar et al., chronotype determined which window participants could receive, and attrition was three times higher in the later-window group [22]. In Dhieb et al., participants chose their own window, and several values in the early-versus-late comparison duplicated other table entries [40]. By contrast, the trials with DXA- or MRI-assessed fat mass did not detect a timing difference [24,25]. The current evidence, therefore, supports an eTRE advantage for body weight in some settings more convincingly than an advantage for fat mass specifically. Durability is also uncertain: in the only trial with post-intervention follow-up, adherence fell to 8–18% within 6 months without dietary guidance, and earlier improvements were partly reversed [37].

#### 4.2.3. Exercise and Lean Mass

Across studies, adding TRE to training was associated with additional fat loss with early [50], later [51,52], and self-selected [34] windows, consistent with meta-analyses showing small additional fat-mass reductions when TRE is combined with exercise [27,28]. It is tempting to conclude that training reduces the influence of window timing and protects lean mass, because the later windows associated with lean-mass loss without exercise [44] were not accompanied by detected lean-mass loss with resistance training [47,51,52]. However, these are comparisons across different studies and populations, the lean-mass comparisons were small and imprecise, and the two direct comparisons under matched training did not report between-group estimates [36,46]. The contrast within Cui et al., in which fat-free mass decreased by 2.3 kg with TRE alone but changed by +0.2 kg with TRE plus resistance training, is the closest within-study evidence, but it was measured by BIA without intake data, and the TRE-plus-training group gained less fat-free mass than the training-only group (+1.6 kg) [34]. The within-group lean-mass decrease with ETRE-PA [36] also shows that training does not prevent lean-mass loss in every setting. These observations are, therefore, treated as hypotheses. Because DXA- and BIA-derived lean mass partly reflects hydration and glycogen [54], modest decreases, including the reduction in appendicular lean mass index in the TREAT trial [44], should also not be equated with loss of contractile tissue.

### 4.3. Protein Intake and Its Distribution Across Meals

Protein distribution is one plausible reason why lean-mass responses might differ between TRE schedules. A shorter window reduces the number of eating occasions, and the meals that are removed differ by timing: eTRE typically removes the evening meal, whereas later windows typically remove breakfast. In controlled studies, spreading protein across several meals increased 24-h or post-exercise muscle protein synthesis compared with a skewed or less frequent pattern [55,56]. More recent data, however, indicate that a single large protein dose can produce a larger and more prolonged anabolic response than previously assumed [57], so the importance of distribution relative to total daily intake remains uncertain.

None of the included studies reported how much protein was consumed at each meal, so this question cannot be addressed directly from the primary evidence. Daily protein intake, where reported, ranged from approximately 0.8–0.9 g/kg/day [52], below the 1.4–2.0 g/kg/day recommended for exercising individuals [58], to approximately 1.6–1.9 g/kg/day [47,51], around the intake above which further gains in resistance-training-induced lean mass are unlikely [59]. Two of the latter trials also provided whey protein around training [47,51]. Supplementary evidence illustrates how distribution can change under TRE. In a single-arm study of a self-selected 8-h window, breakfast was eaten on only about 4 of 28 days, and protein intake was concentrated at lunch and dinner [31]. In a 10-day trial with provided meals and protein spread across three meals within an 8-h window, daily myofibrillar protein synthesis did not differ from a 12-h window, although DXA-assessed lean mass decreased more with TRE (−1.0 vs. −0.2 kg), mainly in the trunk [16]. Future trials should report protein intake by meal and its timing relative to training, so that the contributions of total intake and distribution can be separated from window timing.

### 4.4. Meal Timing, Exercise Timing, and Recovery Feeding

Under TRE, the timing of exercise relative to the eating window determines whether a meal can follow training. This is particularly relevant to eTRE, because afternoon or evening training then falls after the window has closed, and the next opportunity to eat may be 12–16 h later. In four included studies, training took place at or after the end of the window [34,36,46,50]. In Miladi et al., both the early and late groups trained at the end of their windows, at 16:00 and 20:00, respectively, so neither could eat after training, and the timing contrast was confounded with a 4-h difference in exercise clock time [36]. In our 8-week trial, both TRE groups trained at 19:00–20:00 after their windows had closed, and push-up performance and triceps thickness improved to a similar extent as in the control group, which could eat after training; whole-body composition and intake were not measured [46]. Three of these studies reported greater fat loss with TRE plus training, but they also included the lean-mass decrease with ETRE-PA [36] and the smaller fat-free-mass gain with TRE plus resistance training than with training alone [34]. By contrast, training occurred within the window or in the fed state in the studies by Moro et al. (2016), Kotarsky et al., and Tinsley et al. (2019), and two of these provided protein after or around training [47,51,52]. Taken together, these cross-study patterns neither demonstrate nor exclude a role for recovery feeding.

Sports-nutrition guidelines emphasize protein intake in the hours after resistance exercise and carbohydrate intake when glycogen must be restored before the next demanding session [26,60,61]. When only one session is performed per day and total daily intake is adequate, a delay in post-exercise feeding may matter less, because glycogen can be restored within 24 h [61]. The Training–Fuel Coupling framework proposes that training in a low-energy or low-glycogen state and refeeding afterward act as linked phases of adaptation [62]. It provides a useful hypothesis-generating perspective for TRE, in which the window determines whether training is followed by refeeding, but it has not been tested in TRE trials. In practice, the eating window can be positioned so that the main training session falls within it or shortly before a meal: earlier windows suit morning or midday training, and later windows suit evening training. Trials that cross window timing with exercise timing, while recording post-exercise intake, are needed to determine whether this alignment affects adaptation.

### 4.5. Interpretation of Exercise-Performance Findings

#### 4.5.1. Maintaining Versus Improving Performance

The performance data address two different questions. In previously untrained or recreationally active participants starting a new program, the question is whether TRE alters adaptation to training. Strength and muscular endurance improved in most trained groups regardless of window [36,46,47,49,51], but between-condition estimates with confidence intervals were not reported. One trial also detected a group × time interaction in which knee-extensor torque increased with normal eating and decreased with lTRE [52]. These data show that adaptation occurred under TRE but cannot establish that it was equal to adaptation under normal eating.

In athletes who continued their habitual training, the question is whether existing performance is maintained during TRE. The two available studies lasted 4 weeks per condition and included 8 cyclists per group [33] and 15 runners in a crossover [39]. Neither detected a significant change in the main performance outcome, but neither compared early and later windows, and both were too small to exclude decrements of practical importance.

#### 4.5.2. Different Aerobic Outcomes

The aerobic outcomes in this review measure different aspects of performance, and their implications for trained athletes differ. The 6-min walk test reflects functional capacity and was used in sedentary women with overweight or obesity, in whom the improvement reflected training rather than timing [36]; it has little relevance to athletic performance. Time to exhaustion at a fixed submaximal workload [21] and peak oxygen uptake [24] were assessed in non-athletes, and peak oxygen uptake describes aerobic capacity rather than performance. Only the 10-km time trial in runners [39] and maximal oxygen uptake in elite cyclists [33] came from trained athletes. In the time trial, the mean difference in change between conditions was about 16 s (approximately 0.5% of finishing time), but the standard deviations of change were about 3–3.5 min, and the test was run fasted after a graded test. In cyclists, absolute maximal oxygen uptake decreased by 0.24 L/min with TRE and by 0.05 L/min with habitual eating, a difference that was not significant with eight participants per group [33]. The increase in peak power relative to body mass in that study coincided with body-mass loss rather than with higher absolute power. TRE may, therefore, improve power-to-mass ratio in weight-sensitive sports through weight loss, but this possibility depends on whether the energy deficit can be sustained without compromising training (Section 4.7).

#### 4.5.3. The Anaerobic-Power Finding

In our crossover trial, sprint peak power was higher after dTRE than after eTRE by 14.18 W (95% CI, 1.79 to 26.57), whereas body weight decreased more during eTRE [21]. Three explanations other than a lasting adaptation to dTRE need to be considered.

The first is feeding status on the test day. Testing took place between 15:00 and 17:00. During eTRE, this was at or just after the close of the window, when participants had consumed their whole daily intake; during dTRE, it was in the middle of the window, after a shorter feeding period. Because the amount, composition, and timing of the pre-test meals were not reported, it is not possible to determine whether acute nutritional state favored either condition. An explanation based on greater carbohydrate availability before testing under dTRE is, therefore, not supported by the available data.

The second is familiarization. Peak power increased after both conditions although participants did not train, which suggests that one familiarization session did not eliminate learning effects.

The third is order. Sequences were allocated by simple randomization, the number of participants in each sequence was not reported, no new baseline was measured after the washout, and the analysis did not include period or sequence terms. If learning continued across the three test sessions, the condition tested last would be favored, and an imbalance between sequences could produce a difference between conditions.

Time of day alone is unlikely to explain the result, because both conditions were tested during the same afternoon period. However, an interaction between meal timing and the circadian profile of neuromuscular performance cannot be excluded [63,64]. What the trial can establish is that afternoon sprint power was higher after a 4-week dTRE period than after a 4-week eTRE period under the conditions tested. It cannot establish whether this reflects a training-independent adaptation, test-day nutrition, learning, or order. A confirmatory trial would require a standardized pre-test meal, several familiarization sessions, a new baseline before each period, counterbalanced sequences with period-adjusted analysis, and ideally a non-TRE control. Whether training modifies this effect cannot be judged from the present evidence, because the training studies used different tests and populations [36,47].

### 4.6. Mechanistic Considerations

Circadian regulation of metabolism provides a plausible basis for metabolic advantages of earlier eating. Glucose tolerance, pancreatic β-cell responsivity, and insulin sensitivity are generally more favorable during the biological morning than in the evening [65,66,67], and diet-induced thermogenesis after breakfast was approximately 2.5-fold higher than after an equivalent-calorie dinner in a controlled crossover study [17]. Food intake also acts as a timing signal for peripheral metabolic clocks; delaying meals by 5 h shifted the rhythms of plasma glucose and adipose-tissue *PER2* expression without shifting melatonin or cortisol rhythms [64]. These mechanisms could contribute to differences in insulin sensitivity or body weight, but the included trials could not separate them from differences in energy intake, and their magnitude relative to that of an energy deficit is unknown.

Acute differences in substrate use do not necessarily translate into different chronic adaptations. Training in the fed or fasted state within individualized TRE did not produce a significant difference in improvements in fat-oxidation capacity [42], which is consistent with the view that the training stimulus, rather than the feeding context of individual sessions, dominates longer-term adaptation [68,69]. This null comparison was small, however, and does not establish that feeding context is irrelevant.

Chronotype may further modify responses to prescribed eating windows. Evening chronotypes generally eat later, and greater social jetlag has been associated with less favorable obesity-related and metabolic outcomes [70,71]. No included trial randomized participants to early and later windows within chronotype strata, and the one study that incorporated chronotype into allocation could not separate timing from chronotype-related differences [22]. Unmeasured variation in chronotype may, therefore, account for part of the heterogeneity across studies.

### 4.7. Energy and Carbohydrate Availability in Athletes

Short-term data on performance during TRE provide limited information about recovery under sustained high training loads. The two athlete studies lasted 4 weeks per condition [33,39]. In elite cyclists training approximately 500 km/week, body mass decreased by 1.26 kg in 4 weeks despite a prescribed intake of 4800 kcal/day, and intake during the intervention was not recorded [33]. This suggests that an energy deficit occurred during heavy training, but whether energy availability fell below recommended levels cannot be determined.

Meeting energy and carbohydrate needs within a restricted window may become difficult as training load increases. Athletes in heavy training may require 6–10 g/kg/day of carbohydrate or more [60,61], which requires large meals when intake is confined to 8 h. Morning sessions before the window opens, twice-daily training, and evening competitions further limit the scope for fueling and recovery. Sustained low energy availability is associated with impaired health and performance [72]. In addition, both studies of athletes maintaining habitual endurance training enrolled only men, and no included study assessed markers of low energy availability. The present findings, therefore, cannot be extended to longer training blocks, competition periods, or female athletes. If TRE is used during training, energy intake, body mass, training quality, and indicators of low energy availability should be monitored.

### 4.8. Practical Implications

The evidence does not support prescribing one TRE schedule universally, and recommendations should be read as cautious. For individuals seeking weight loss without deliberate calorie counting, an earlier window may be tried if it suits daily routines, but the largest trial found no advantage over a later window [25], so the choice can reasonably be based on feasibility. When a defined energy deficit is followed, the window can likewise be chosen for adherence, provided that achieved intake is checked against the prescription. Work schedules and family commitments were among the barriers to TRE reported in a short-term study [30], and adherence declined substantially after supervision ended [37]. For individuals seeking fat loss while preserving lean mass, general sports-nutrition evidence supports resistance training, adequate daily protein intake, and protein-containing meals distributed within the window, including one after training [58,59]; these recommendations are not specific to TRE trials. For athletes, the window should, where possible, be positioned so that key sessions are followed by a meal, and TRE should be used cautiously during periods of heavy training or competition [60,72].

### 4.9. Limitations

Although 23 studies were included, only eight directly compared earlier and later windows. These eight differed in energy prescription, exercise exposure, population, and timing definitions; one compared eTRE with a midday window, one allocated windows by chronotype, and one allowed participants to choose their window. Fourteen studies were rated lower in confidence, most had small samples and interventions of 12 weeks or less, and several relied on completer or per-protocol analyses. Body composition was assessed using DXA, BIA, MRI, anthropometry, or localized ultrasound [54]. Energy intake was measured in only 14 studies, no study provided all meals, and no study reported protein intake by meal. Populations ranged from healthy young adults and trained athletes to sedentary, older, and overweight or obese adults; several studies enrolled only one sex, the two athlete studies enrolled only men, and few studies were powered to examine sex- or chronotype-specific responses. Few studies reported between-condition estimates with confidence intervals, which limited our ability to distinguish inconclusive findings from evidence of no difference. Two of the included trials were conducted by the present authors; their findings were appraised using the same criteria applied to the other studies, and the limitations of one of them are discussed in detail above. Study selection, data extraction, and methodological appraisal were performed by a single reviewer. The appraisal used a pragmatic rule rather than a validated risk-of-bias tool, the Google Scholar search was limited to the first 100 results of each sub-query, and one eligible trial was missed during the original screening and added during revision. Effect sizes were not pooled. The conclusions should, therefore, be interpreted as a structured assessment of the direction, consistency, and certainty of the evidence rather than as definitive estimates of the comparative efficacy of eTRE and dTRE.

### 4.10. Future Research

Three hypotheses generated by this review require direct testing: that energy control modifies the effect of window timing, that structured training reduces it, and that timing affects lean mass through protein distribution or recovery feeding. Factorial trials crossing window timing with controlled or verified energy intake, and with matched training scheduled at fixed clock times, would test the first two. Trials should report achieved energy and protein intake by meal, objectively verified meal timing, the timing of exercise relative to meals, and between-condition estimates with confidence intervals. Crossover trials of performance should standardize pre-test meals, include multiple familiarization sessions, re-establish baseline before each period, and use counterbalanced sequences. Finally, longer studies in trained athletes of both sexes, conducted during periods of heavy training and with monitoring of energy availability, are needed before TRE can be recommended for athletic populations.

## 5. Conclusions

Evidence from 23 intervention studies does not show that earlier TRE is consistently better than later TRE for body composition or exercise performance. No direct comparison favored the later window for body composition, but the advantages reported for the earlier window came from a small crossover trial and two non-randomized comparisons, and the randomized parallel-group trials, including the largest, did not detect a difference. When TRE was combined with structured training, additional fat loss was observed with early, later, and self-selected windows, but whether training reduces the influence of timing or protects lean mass remains a hypothesis. No direct comparison detected an early-versus-late difference in strength, muscular endurance, or aerobic outcomes; these imprecise findings do not establish that performance is maintained, particularly in trained athletes during sustained heavy training. The greater sprint-power response to dTRE in one trial may reflect test-day feeding, learning, or order effects and requires confirmation in a trial designed to control them. In practice, the eating window can be chosen primarily for feasibility and adherence, positioned where possible so that key training sessions are followed by a meal, and combined with resistance training and adequate energy and protein intake. Future trials should cross window timing with controlled or verified energy intake and with matched training at fixed clock times, report protein intake by meal and between-condition estimates with confidence intervals, and stratify participants by chronotype.

## Data Availability

No new data were created or analyzed in this study. Data sharing is not applicable to this article.

## Supplementary Materials

The following supporting information can be downloaded at: https://www.mdpi.com/article/10.3390/nu18213603/s1, Table S1: Google Scholar sub-queries, search dates, and records retrieved and screened; Table S2: Reports retained as narrative supplementary evidence and the eligibility criterion not met; Table S3: Study-level methodological appraisal of the 23 primary studies.

## Abbreviations

The following abbreviations are used in this manuscript:

1RM	one-repetition maximum
ANCOVA	analysis of covariance
BIA	bioelectrical impedance analysis
CCR	continuous caloric restriction
CI	confidence interval
CR	caloric restriction
dTRE	delayed time-restricted eating
DXA	dual-energy X-ray absorptiometry
ER	energy restriction
eTRE	early time-restricted eating
ETRE-PA	early time-restricted eating combined with physical activity
eTRF	early time-restricted feeding
Fatmax	exercise intensity eliciting maximal fat oxidation
HIFT	high-intensity functional training
HMB	β-hydroxy-β-methylbutyrate
IF	intermittent fasting
ITT	intention-to-treat
lTRE	late time-restricted eating
LTRE-PA	late time-restricted eating combined with physical activity
mTRE	midday time-restricted eating
mTRF	midday time-restricted feeding
MRI	magnetic resonance imaging
PA	physical activity
PRISMA	Preferred Reporting Items for Systematic Reviews and Meta-Analyses
RCT	randomized controlled trial
RT	resistance training
TRE	time-restricted eating
TRF	time-restricted feeding
UC	usual care
VO_2max_	maximal oxygen uptake

## References

[1] World Health Organization (2025). Obesity and Overweight. https://www.who.int/news-room/fact-sheets/detail/obesity-and-overweight.

[2] Phelps N.H., Singleton R.K., Zhou B., Heap R.A., Mishra A., Bennett J.E., Paciorek C.J., Lhoste V.P., Carrillo-Larco R.M., Stevens G.A. (2024). Worldwide trends in underweight and obesity from 1990 to 2022: A pooled analysis of 3663 population-representative studies with 222 million children, adolescents, and adults. Lancet.

[3] Brauer M., Roth G.A., Aravkin A.Y., Zheng P., Abate K.H., Abate Y.H., Abbafati C., Abbasgholizadeh R., Abbasi M.A., Abbasian M. (2024). Global burden and strength of evidence for 88 risk factors in 204 countries and 811 subnational locations, 1990–2021: A systematic analysis for the Global Burden of Disease Study 2021. Lancet.

[4] Powell-Wiley T.M., Poirier P., Burke L.E., Després J.P., Gordon-Larsen P., Lavie C.J., Lear S.A., Ndumele C.E., Neeland I.J., Sanders P. (2021). Obesity and cardiovascular disease: A scientific statement from the American Heart Association. Circulation.

[5] Headland M., Clifton P.M., Carter S., Keogh J.B. (2016). Weight-loss outcomes: A systematic review and meta-analysis of intermittent energy restriction trials lasting a minimum of 6 months. Nutrients.

[6] Machado A.M., Guimarães N.S., Bocardi V.B., da Silva T.P.R., do Carmo A.S., de Menezes M.C., Duarte C.K. (2022). Understanding weight regain after a nutritional weight loss intervention: Systematic review and meta-analysis. Clin. Nutr. ESPEN.

[7] Ezpeleta M., Cienfuegos S., Lin S., Pavlou V., Gabel K., Tussing-Humphreys L., Varady K.A. (2024). Time-restricted eating: Watching the clock to treat obesity. Cell Metab..

[8] Koppold D.A., Breinlinger C., Hanslian E., Kessler C., Cramer H., Khokhar A.R., Peterson C.M., Tinsley G., Vernieri C., Bloomer R.J. (2024). International consensus on fasting terminology. Cell Metab..

[9] Varady K.A., Cienfuegos S., Ezpeleta M., Gabel K. (2022). Clinical application of intermittent fasting for weight loss: Progress and future directions. Nat. Rev. Endocrinol..

[10] Adafer R., Messaadi W., Meddahi M., Patey A., Haderbache A., Bayen S., Messaadi N. (2020). Food timing, circadian rhythm and chrononutrition: A systematic review of time-restricted eating’s effects on human health. Nutrients.

[11] Liu H.Y., Eso A.A., Cook N., O’Neill H.M., Albarqouni L. (2024). Meal timing and anthropometric and metabolic outcomes: A systematic review and meta-analysis. JAMA Netw. Open.

[12] Liu L., Chen W., Wu D., Hu F. (2022). Metabolic efficacy of time-restricted eating in adults: A systematic review and meta-analysis of randomized controlled trials. J. Clin. Endocrinol. Metab..

[13] Semnani-Azad Z., Khan T.A., Chiavaroli L., Chen V., Bhatt H.A., Chen A., Chiang N., Oguntala J., Kabisch S., Lau D.C. (2025). Intermittent fasting strategies and their effects on body weight and other cardiometabolic risk factors: Systematic review and network meta-analysis of randomised clinical trials. BMJ.

[14] Longo V.D., Panda S. (2016). Fasting, circadian rhythms, and time-restricted feeding in healthy lifespan. Cell Metab..

[15] Sutton E.F., Beyl R., Early K.S., Cefalu W.T., Ravussin E., Peterson C.M. (2018). Early time-restricted feeding improves insulin sensitivity, blood pressure, and oxidative stress even without weight loss in men with prediabetes. Cell Metab..

[16] Parr E.B., Kouw I.W., Wheeler M.J., Radford B.E., Hall R.C., Senden J.M., Goessens J.P., Van Loon L.J., Hawley J.A. (2023). Eight-hour time-restricted eating does not lower daily myofibrillar protein synthesis rates: A randomized control trial. Obesity.

[17] Richter J., Herzog N., Janka S., Baumann T., Kistenmacher A., Oltmanns K.M. (2020). Twice as high diet-induced thermogenesis after breakfast vs dinner on high-calorie as well as low-calorie meals. J. Clin. Endocrinol. Metab..

[18] Manoogian E.N., Chow L.S., Taub P.R., Laferrère B., Panda S. (2022). Time-restricted eating for the prevention and management of metabolic diseases. Endocr. Rev..

[19] Liu J., Yi P., Liu F. (2023). The effect of early time-restricted eating vs later time-restricted eating on weight loss and metabolic health. J. Clin. Endocrinol. Metab..

[20] Petridi F., Geurts J.M., Nyakayiru J., Schaafsma A., Schaafsma D., Meex R.C., Singh-Povel C.M. (2024). Effects of Early and Late Time-Restricted Feeding on Parameters of Metabolic Health: An Explorative Literature Assessment. Nutrients.

[21] Yu Z., Ueda T. (2025). Time-restricted eating without exercise enhances anaerobic power and reduces body weight: A randomized crossover trial in untrained adults. Nutrients.

[22] Črešnovar T., Habe B., Mohorko N., Kenig S., Pražnikar Z.J., Petelin A. (2025). Early time-restricted eating with energy restriction has a better effect on body fat mass, diastolic blood pressure, metabolic age and fasting glucose compared to late time-restricted eating with energy restriction and/or energy restriction alone: A 3-month randomized clinical trial. Clin. Nutr..

[23] Xie Z., Sun Y., Ye Y., Hu D., Zhang H., He Z., Zhao H., Yang H., Mao Y. (2022). Randomized controlled trial for time-restricted eating in healthy volunteers without obesity. Nat. Commun..

[24] do Nascimento Queiroz J., Macedo R.C.O., dos Santos G.C., Munhoz S.V., Machado C.L.F., de Menezes R.L., Menzem E.N., Moritz C.E.J., Pinto R.S., Tinsley G.M. (2023). Cardiometabolic Effects of Early versus Delayed Time-Restricted Eating Plus Energetic Restriction in Adults with Overweight and Obesity: An Exploratory Randomised Clinical Trial. Br. J. Nutr..

[25] Dote-Montero M., Clavero-Jimeno A., Merchan-Ramirez E., Oses M., Echarte J., Camacho-Cardenosa A., Concepcion M., Amaro-Gahete F.J., Alcántara J.M., Lopez-Vazquez A. (2025). Effects of early, late and self-selected time-restricted eating on visceral adipose tissue and cardiometabolic health in participants with overweight or obesity: A randomized controlled trial. Nat. Med..

[26] Kerksick C.M., Arent S., Schoenfeld B.J., Stout J.R., Campbell B., Wilborn C.D., Taylor L., Kalman D., Smith-Ryan A.E., Kreider R.B. (2017). International Society of Sports Nutrition Position Stand: Nutrient Timing. J. Int. Soc. Sports Nutr..

[27] Hays H.M., Sefidmooye Azar P., Kang M., Tinsley G.M., Wijayatunga N.N. (2025). Effects of Time-Restricted Eating with Exercise on Body Composition in Adults: A Systematic Review and Meta-Analysis. Int. J. Obes..

[28] Wan K.W., Dai Z.H., Ho R.S.T., Huang W.Y., Wong S.H.S. (2024). Comparative Effects of Time-Restricted Feeding versus Normal Diet on Physical Performance and Body Composition in Healthy Adults with Regular Exercise Habits: A Systematic Review and Meta-Analysis. BMJ Open Sport Exerc. Med..

[29] Allison K.C., Hopkins C.M., Ruggieri M., Spaeth A.M., Ahima R.S., Zhang Z., Taylor D.M., Goel N. (2021). Prolonged, Controlled Daytime versus Delayed Eating Impacts Weight and Metabolism. Curr. Biol..

[30] Parr E.B., Devlin B.L., Radford B.E., Hawley J.A. (2020). A Delayed Morning and Earlier Evening Time-Restricted Feeding Protocol for Improving Glycemic Control and Dietary Adherence in Men with Overweight/Obesity: A Randomized Controlled Trial. Nutrients.

[31] Park S.J., Yang J.W., Song Y.J. (2021). The Effect of Four Weeks Dietary Intervention with 8-Hour Time-Restricted Eating on Body Composition and Cardiometabolic Risk Factors in Young Adults. Nutrients.

[32] Čermáková E., Forejt M., Čermák M. (2024). The Influence of Intermittent Fasting on Selected Human Anthropometric Parameters. Int. J. Med. Sci..

[33] Moro T., Tinsley G., Longo G., Grigoletto D., Bianco A., Ferraris C., Guglielmetti M., Veneto A., Tagliabue A., Marcolin G. (2020). Time-restricted eating effects on performance, immune function, and body composition in elite cyclists: A randomized controlled trial. J. Int. Soc. Sports Nutr..

[34] Cui T., Sun Y., Ye W., Liu Y., Korivi M. (2025). Efficacy of time restricted eating and resistance training on body composition and mood profiles among young adults with overweight/obesity: A randomized controlled trial. J. Int. Soc. Sports Nutr..

[35] Miladi S., Hammouda O., Miladi Trabelsi S.J., Miladi S.C., Ameur R., Driss T. (2026). The Combination of Physical Activity and Late-Time-Restricted Eating Improves Eating Behavior, Cognitive Performance and Cardiovascular Parameters in Women Living with Excess Body Weight. Physiol. Behav..

[36] Miladi S., Driss T., Ameur R., Miladi S.C., Miladi S.J., Najjar M.F., Neffati F., Hammouda O. (2025). Effectiveness of early versus late time-restricted eating combined with physical activity in overweight or obese women. Nutrients.

[37] Črešnovar T., Habe B., Mohorko N., Kenig S., Jenko Pražnikar Z., Petelin A. (2026). Adherence and Metabolic Outcomes of Early and Late Time-Restricted Eating with Energy Restriction vs. Energy Restriction Alone: A 6-Month Follow-Up. Nutrients.

[38] Richardson C.E., Tovar A.P., Davis B.A., Van Loan M.D., Keim N.L., Casazza G.A. (2023). An Intervention of Four Weeks of Time-Restricted Eating (16/8) in Male Long-Distance Runners Does Not Affect Cardiometabolic Risk Factors. Nutrients.

[39] Tovar A.P., Richardson C.E., Keim N.L., Van Loan M.D., Davis B.A., Casazza G.A. (2021). Four Weeks of 16/8 Time Restrictive Feeding in Endurance Trained Male Runners Decreases Fat Mass, without Affecting Exercise Performance. Nutrients.

[40] Dhieb N., Othman R.B., Karmous I., Mizouri R., Nouira M., Kacem A., Gamoudi A., Douiri W., Mansour N.B., Jamoussi H. (2025). Comparative Effects of 16: 8 Intermittent Fasting and Continuous Caloric Restriction on Metabolic Syndrome Components in obese Women: A Non-randomized Controlled Clinical Trial. La Tunis. Méd..

[41] Sampieri A., Paoli A., Spinello G., Santinello E., Moro T. (2024). Impact of daily fasting duration on body composition and cardiometabolic risk factors during a time-restricted eating protocol: A randomized controlled trial. J. Transl. Med..

[42] Hofstätter F., Niedermeier M., Rausch L.K., Kopp M., Simpson L., Lawley J.S. (2025). Effects of Time-Restricted Feeding and Meal Timing on an 8-Week Fat Oxidation Exercise Training Program—A Randomized Controlled Trial. Physiol. Rep..

[43] Mena-Hernández D.R., Jiménez-Domínguez G., Méndez J.D., Olvera-Hernández V., Martínez-López M.C., Guzmán-Priego C.G., Reyes-López Z., Ramos-García M., Juárez-Rojop I.E., Zavaleta-Toledo S.S. (2024). Effect of early time-restricted eating on metabolic markers and body composition in individuals with overweight or obesity. Nutrients.

[44] Lowe D.A., Wu N., Rohdin-Bibby L., Moore A.H., Kelly N., Liu Y.E., Philip E., Vittinghoff E., Heymsfield S.B., Olgin J.E. (2020). Effects of time-restricted eating on weight loss and other metabolic parameters in women and men with overweight and obesity: The TREAT randomized clinical trial. JAMA Intern. Med..

[45] Domaszewski P., Konieczny M., Pakosz P., Łukaniszyn-Domaszewska K., Mikuláková W., Sadowska-Krępa E., Anton S. (2022). Effect of a Six-Week Times Restricted Eating Intervention on the Body Composition in Early Elderly Men with Overweight. Sci. Rep..

[46] Yu Z., Ueda T. (2025). Early Time-Restricted Eating Improves Weight Loss While Preserving Muscle: An 8-Week Trial in Young Women. Nutrients.

[47] Tinsley G.M., Moore M.L., Graybeal A.J., Paoli A., Kim Y., Gonzales J.U., Harry J.R., VanDusseldorp T.A., Kennedy D.N., Cruz M.R. (2019). Time-Restricted Feeding Plus Resistance Training in Active Females: A Randomized Trial. Am. J. Clin. Nutr..

[48] Jamshed H., Steger F.L., Bryan D.R., Richman J.S., Warriner A.H., Hanick C.J., Martin C.K., Salvy S.J., Peterson C.M. (2022). Effectiveness of early time-restricted eating for weight loss, fat loss, and cardiometabolic health in adults with obesity: A randomized clinical trial. JAMA Intern. Med..

[49] Tinsley G.M., Forsse J.S., Butler N.K., Paoli A., Bane A.A., La Bounty P.M., Morgan G.B., Grandjean P.W. (2017). Time-Restricted Feeding in Young Men Performing Resistance Training: A Randomized Controlled Trial. Eur. J. Sport Sci..

[50] Ameur R., Maaloul R., Tagougui S., Neffati F., Hadj Kacem F., Najjar M.F., Ammar A., Hammouda O. (2024). Unlocking the power of synergy: High-intensity functional training and early time-restricted eating for transformative changes in body composition and cardiometabolic health in inactive women with obesity. PLoS ONE.

[51] Moro T., Tinsley G., Bianco A., Marcolin G., Pacelli Q.F., Battaglia G., Palma A., Gentil P., Neri M., Paoli A. (2016). Effects of eight weeks of time-restricted feeding (16/8) on basal metabolism, maximal strength, body composition, inflammation, and cardiovascular risk factors in resistance-trained males. J. Transl. Med..

[52] Kotarsky C.J., Johnson N.R., Mahoney S.J., Mitchell S.L., Schimek R.L., Stastny S.N., Hackney K.J. (2021). Time-restricted eating and concurrent exercise training reduces fat mass and increases lean mass in overweight and obese adults. Physiol. Rep..

[53] Thomas E.A., Zaman A., Sloggett K.J., Steinke S., Grau L., Catenacci V.A., Cornier M.A., Rynders C.A. (2022). Early time-restricted eating compared with daily caloric restriction: A randomized trial in adults with obesity. Obesity.

[54] Marra M., Sammarco R., De Lorenzo A., Iellamo F., Siervo M., Pietrobelli A., Donini L.M., Santarpia L., Cataldi M., Pasanisi F. (2019). Assessment of Body Composition in Health and Disease Using Bioelectrical Impedance Analysis and Dual Energy X-Ray Absorptiometry: A Critical Overview. Contrast Media Mol. Imaging.

[55] Areta J.L., Burke L.M., Ross M.L., Camera D.M., West D.W.D., Broad E.M., Jeacocke N.A., Moore D.R., Stellingwerff T., Phillips S.M. (2013). Timing and Distribution of Protein Ingestion during Prolonged Recovery from Resistance Exercise Alters Myofibrillar Protein Synthesis. J. Physiol..

[56] Mamerow M.M., Mettler J.A., English K.L., Casperson S.L., Arentson-Lantz E., Sheffield-Moore M., Layman D.K., Paddon-Jones D. (2014). Dietary Protein Distribution Positively Influences 24-h Muscle Protein Synthesis in Healthy Adults. J. Nutr..

[57] Trommelen J., van Lieshout G.A.A., Nyakayiru J., Holwerda A.M., Smeets J.S.J., Hendriks F.K., van Kranenburg J.M.X., Zorenc A.H., Senden J.M., Goessens J.P.B. (2023). The Anabolic Response to Protein Ingestion during Recovery from Exercise Has No Upper Limit in Magnitude and Duration in Vivo in Humans. Cell Rep. Med..

[58] Jäger R., Kerksick C.M., Campbell B.I., Cribb P.J., Wells S.D., Skwiat T.M., Purpura M., Ziegenfuss T.N., Ferrando A.A., Arent S.M. (2017). International Society of Sports Nutrition Position Stand: Protein and Exercise. J. Int. Soc. Sports Nutr..

[59] Morton R.W., Murphy K.T., McKellar S.R., Schoenfeld B.J., Henselmans M., Helms E., Aragon A.A., Devries M.C., Banfield L., Krieger J.W. (2018). A Systematic Review, Meta-Analysis and Meta-Regression of the Effect of Protein Supplementation on Resistance Training-Induced Gains in Muscle Mass and Strength in Healthy Adults. Br. J. Sports Med..

[60] Thomas D.T., Erdman K.A., Burke L.M. (2016). Nutrition and Athletic Performance. Med. Sci. Sports Exerc..

[61] Burke L.M., Hawley J.A., Wong S.H.S., Jeukendrup A.E. (2011). Carbohydrates for Training and Competition. J. Sports Sci..

[62] Stoian M., Mănescu D.C. (2026). Training–Fuel Coupling (TFC): A Molecular Sports Nutrition Framework for Energy Availability, Chrono-Nutrition, and Performance Optimization. Nutrients.

[63] Ravindrakumar A., Bommasamudram T., Tod D., Edwards B.J., Chtourou H., Pullinger S.A. (2022). Daily Variation in Performance Measures Related to Anaerobic Power and Capacity: A Systematic Review. Chronobiol. Int..

[64] Wehrens S.M.T., Christou S., Isherwood C., Middleton B., Gibbs M.A., Archer S.N., Skene D.J., Johnston J.D. (2017). Meal Timing Regulates the Human Circadian System. Curr. Biol..

[65] Poggiogalle E., Jamshed H., Peterson C.M. (2018). Circadian Regulation of Glucose, Lipid, and Energy Metabolism in Humans. Metabolism.

[66] Morris C.J., Yang J.N., Garcia J.I., Myers S., Bozzi I., Wang W., Buxton O.M., Shea S.A., Scheer F.A.J.L. (2015). Endogenous Circadian System and Circadian Misalignment Impact Glucose Tolerance via Separate Mechanisms in Humans. Proc. Natl. Acad. Sci. USA.

[67] Saad A., Dalla Man C., Nandy D.K., Levine J.A., Bharucha A.E., Rizza R.A., Basu R., Carter R.E., Cobelli C., Kudva Y.C. (2012). Diurnal Pattern to Insulin Secretion and Insulin Action in Healthy Individuals. Diabetes.

[68] Vieira A.F., Costa R.R., Macedo R.C.O., Coconcelli L., Kruel L.F.M. (2016). Effects of Aerobic Exercise Performed in Fasted versus Fed State on Fat and Carbohydrate Metabolism in Adults: A Systematic Review and Meta-Analysis. Br. J. Nutr..

[69] Aird T.P., Davies R.W., Carson B.P. (2018). Effects of Fasted vs Fed-State Exercise on Performance and Post-Exercise Metabolism: A Systematic Review and Meta-Analysis. Scand. J. Med. Sci. Sports.

[70] Teixeira G.P., Guimarães K.C., Soares A.G.N.S., Marqueze E.C., Moreno C.R.C., Mota M.C., Crispim C.A. (2022). Role of Chronotype in Dietary Intake, Meal Timing, and Obesity: A Systematic Review. Nutr. Rev..

[71] Lin M.Y., Kang Y.N., Apriliyasari R.W., Tsai P.S. (2024). Association Between Social Jetlag and Components of Metabolic Syndrome: A Systematic Review and Meta-Analysis. J. Nurs. Res..

[72] Mountjoy M., Ackerman K.E., Bailey D.M., Burke L.M., Constantini N., Hackney A.C., Heikura I.A., Melin A., Pensgaard A.M., Stellingwerff T. (2023). 2023 International Olympic Committee’s (IOC) Consensus Statement on Relative Energy Deficiency in Sport (REDs). Br. J. Sports Med..

