# Supplementary material for: Is Earlier Always Better? Early Versus Later Time-Restricted Eating, Body Composition, and Exercise Performance: A Narrative Review
Source: Nutrients. 2026 Oct 30;18(21):3603. doi: 10.3390/nu18213603 (PMC13764830; doi:10.3390/nu18213603)
Supplement: Supplementary file 1 [file nutrients-18-03603-s001.zip › nutrients-4627565-supplementary.pdf]

Supplementary Materials

Is Earlier Always Better? Early versus Later Time-Restricted Eating, Body Composition, and Exercise Performance: A Narrative Review

Zifu Yu, Takeshi Ueda and Pengcheng Zhou

Contents

Table S1. Google Scholar sub-queries, search dates, and records retrieved and screened.
Table S2. Reports retained as narrative supplementary evidence and the eligibility criterion not met.
Table S3. Study-level methodological appraisal of the 23 primary studies.

Numbers in square brackets refer to the reference list of the main text.

Table S1. Google Scholar sub-queries, search dates, and records retrieved and screened

No.	Sub-query	Records retrieved	Records screened	Eligible studies identified	
				18 June 2026	13 October 2026
Q1	`("time-restricted eating" OR "time-restricted feeding" OR "time-restricted diet") (early OR midday OR late OR delayed OR morning OR evening OR timing OR chrononutrition) ("body composition" OR "fat mass" OR "lean mass")`	8,890	100	Queiroz et al. (2023) [24]; Moro et al. (2020) [33]; Cui et al. (2025) [34]	—
Q2	`("time-restricted eating" OR "time-restricted feeding" OR "time-restricted diet") (early OR midday OR late OR delayed OR morning OR evening OR timing OR chrononutrition) ("muscle mass" OR "fat-free mass" OR "body fat")`	7,430	100	Xie et al. (2022) [23]	Dote-Montero et al. (2025) [25]
Q3	`("time-restricted eating" OR "time-restricted feeding" OR "time-restricted diet") (early OR midday OR late OR delayed OR morning OR evening OR timing OR chrononutrition) ("exercise performance" OR "physical performance")`	1,970	100	—	—
Q4	`("time-restricted eating" OR "time-restricted feeding" OR "time-restricted diet") (early OR midday OR late OR delayed OR morning OR evening OR timing OR chrononutrition) ("muscle strength" OR "muscular endurance")`	1,450	100	—	—
Q5	`("time-restricted eating" OR "time-restricted feeding" OR "time-restricted diet") (early OR midday OR late OR delayed OR morning OR evening OR timing OR chrononutrition) ("aerobic capacity" OR "anaerobic power" OR "explosive performance")`	623	100	—	—
Q6	`("intermittent fasting" OR "eating window" OR "16:8") (early OR midday OR late OR delayed OR morning OR evening OR timing OR chrononutrition) ("body composition" OR "fat mass" OR "lean mass")`	24,200	100	—	—
Q7	`("intermittent fasting" OR "eating window" OR "16:8") (early OR midday OR late OR delayed OR morning OR evening OR timing OR chrononutrition) ("muscle mass" OR "fat-free mass" OR "body fat")`	22,500	100	—	—
Q8	`("intermittent fasting" OR "eating window" OR "16:8") (early OR midday OR late OR delayed OR morning OR evening OR timing OR chrononutrition) ("exercise performance" OR "physical performance")`	16,000	100	—	—
Q9	`("intermittent fasting" OR "eating window" OR "16:8") (early OR midday OR late OR delayed OR morning OR evening OR timing OR chrononutrition) ("muscle strength" OR "muscular endurance")`	16,500	100	—	—
Q10	`("intermittent fasting" OR "eating window" OR "16:8") (early OR midday OR late OR delayed OR morning OR evening OR timing OR chrononutrition) ("aerobic capacity" OR "anaerobic power" OR "explosive performance")`	6,300	100	—	—

Notes: Each sub-query combines one group of TRE terms, the full group of timing terms, and one group of outcome terms. Terms within parentheses are combined with OR, and parenthetical groups separated by a space are combined with AND. Together, the 10 sub-queries cover all terms of the PubMed search string (Section 2.2 of the main text) except the abbreviations "TRE" and "TRF", which were omitted to avoid irrelevant matches. The search was run on 18 June 2026 and repeated on 13 October 2026 with the same sub-queries and settings; the number of records retrieved for each sub-query, as reported by Google Scholar (an approximate count), was the same on both dates. Results were sorted by relevance, and the first 100 records of each sub-query were screened by title and abstract. "—" indicates that no new eligible study was identified. Only eligible studies not already identified by the initial search (Figure 1 of the main text) are listed; Dote-Montero et al. [25] had been overlooked in the earlier screening.

Table S2. Reports retained as narrative supplementary evidence and the eligibility criterion not met

Report	Design and intervention	Eligibility criterion not met	Use in the review
Allison et al. (2021) [29]	Randomized crossover in 12 adults (BMI 19–27 kg/m^2); all meals provided; daytime (08:00–19:00) vs. delayed (12:00–23:00) eating for 8 weeks each	Eating windows of 11 h exceed the TRE definition (no more than approximately 10 h)	Section 4.2.1: evidence on window timing when energy intake is controlled
Parr et al. (2020) [30]	Randomized crossover in 11 men with overweight or obesity; isoenergetic diets; TRF 8 h/day (meals at 10:00, 13:00, and 17:00) vs. extended feeding 15 h/day, 5 days each	Intervention shorter than four weeks	Section 4.8: barriers to adherence (work schedules, family commitments, social events)
Park et al. (2021) [31]	Single-arm study of a self-selected 8-h window for 4 weeks in 33 young adults; daily dietary records	No comparison condition	Section 4.3: distribution of meals and protein intake under TRE
Čermáková et al. (2024) [32]	Randomized, 3 arms (6-h window plus 25% energy restriction, 25% energy restriction alone, 6-h window without restriction) with self-selected windows; 95 randomized, 75 completed	Intervention of 21 days (shorter than four weeks); clock times of the windows not reported	Not used to support specific statements; listed for completeness

Notes: These four reports were identified at the full-text assessment stage (Figure 1 of the main text) and did not meet one or more eligibility criteria. They were not included in data extraction or in the primary synthesis and were cited only where they aided interpretation. Two further reports considered supplementary in the original submission (Sampieri et al., 2024 [41]; Dhieb et al., 2025 [40]) met all eligibility criteria on re-examination and are now included in the primary synthesis, with their limitations reflected in Table S3.

Table S3. Study-level methodological appraisal of the 23 primary studies

Study [ref.]	Allocation	Participants: entered → analyzed (attrition); attrition by condition	Analysis population	Energy intake (prescribed, assessed, or controlled)	Adherence to the eating window	Consistency of reported data	Confidence (main reason)
**Studies without concurrent structured exercise training (Table 1)**							
Xie et al. (2022) [23]	Randomized parallel (eTRF/mTRF/control, 30 each); outcome assessors and statisticians blinded	90 → 82 (8.9%); eTRF 6.7%, mTRF 13.3%, control 6.7%	Completers	Ad libitum; assessed from meal photographs; change −240 vs. −159 kcal/day (eTRF vs. mTRF; $p = 0.30$), not shown to be equivalent	Photo-verified meal timing; compliant days 96.8% (eTRF) and 98.2% (mTRF)	No key inconsistency; control arm mislabelled in flow diagram	Moderate (completers only; BIA)
Yu and Ueda (2025), crossover [21]	Randomized crossover (simple randomization; sequence counts NR); 2-week washout; no new baseline after washout	28 → 28 (0%)	All participants (linear mixed model); period and sequence terms not included	No target; 3-day photographic records in each phase; intake higher during dTRE (+64 kcal/day; $p = 0.040$)	Weekly check-ins and self-report; adherence rate NR	Reported effect size for energy intake does not match the paired test; peak-power CI reported in Abstract only	Moderate (no period/sequence adjustment; intake differed between conditions)
Črešnovar et al. (2025; 2026 follow-up) [22, 37]	Randomization restricted by chronotype: morning types to eTRE+ER or ER, others to lTRE+ER or ER; eTRE vs. lTRE not randomized	108 → 93 at 3 months (13.9%); eTRE 8.1%, lTRE 24.3%, ER 8.8% (gap 16.2 pp); 69 of 93 at 6-month follow-up	Per protocol (adherent completers); follow-up responders only	ER prescribed (RMR × PAL − 500 kcal/day); 3-day food records; no dietary guidance during follow-up	Diet/time calendar and dietitian meetings; exclusion after 4 non-adherent days; ≥5 adherent days/week at follow-up in 8–18%	Window tolerance described differently in the two reports (1 h vs. ±30 min)	Lower (non-randomized timing contrast; attrition gap >10 pp)
Queiroz et al. (2023) [24]	Randomized parallel (16/16/16), stratified by sex and BMI; data collectors blinded; exploratory, stopped early (COVID-19)	48 → 37 (22.9%); eTRE 19%, dTRE 31%, CR 19% (gap 12.5 pp)	Per protocol in main text; ITT (GEE) in supplement	25% energy deficit prescribed; two 3-day food records; reported intake 59–67% of the prescribed intake target	Real-time meal photographs; fully adherent 85% (eTRE, CR) and 73% (dTRE)	No key inconsistency	Lower (attrition >20% with per-protocol analysis; gap >10 pp)
Dote-Montero et al. (2025) [25]	Randomized parallel, 4 groups, stratified by site and sex	197 → 183 (7.1%); 4.1–10.6% by group	ITT ($n = 197$; linear mixed models)	No target; three 24-h recalls; early − late −143 kcal/day (95% CI −403 to 118)	App-recorded first and last meal; adherent days 85–88%	None identified	Higher
Dhieb et al. (2025) [40]	Non-randomized: participants chose IF or CCR, then an early (08:00–16:00) or late (12:00–20:00) window	100 → 75; IF 33.3%, CCR 18.2% (gap 15.2 pp); early/late completers 16/14	Adherent completers	25% deficit prescribed; achieved intake not quantified	Weekly calls and visits every 2 weeks; adherence defined as ≥5 days/week; achieved adherence NR	Values in the early-vs.-late table duplicate other table entries; sample sizes differ between text and tables	Lower (non-randomized; attrition gap; inconsistent data)
Jamshed et al. (2022) [48]	Randomized parallel (45/45), stratified by sex, race, and physical activity	90 → 70 (22.2% withdrew); control 20.0%, eTRE 24.4%	ITT ($n = 90$; mixed models and multiple imputation)	Both arms prescribed 500 kcal/day below REE; 3-day photographic records; measured difference 1 kcal/day (95% CI −251 to 253); modeled intake 214 kcal/day lower with eTRE	Daily self-reported first and last eating times; 6.0 vs. 6.3 adherent days/week	No key inconsistency; fat-mass advantage significant only in completers	Moderate (attrition >20% but ITT)
Thomas et al. (2022) [53]	Randomized parallel (42/43); 4 withdrew before starting	85 → 63 at week 39 (25.9%); control 26.2%, eTRE 25.6%	ITT-labelled mixed models (81 starters)	Both arms given a personalized calorie goal; 3-day photographic records; week-12 difference $p = 0.26$	Questionnaires and photographic meal-time records; median last meal 18:43 at week 12 (window relative to waking)	Adherence differs between text and table (5.1 vs. 5.5 days/week)	Moderate (attrition >20% with mixed-model analysis)
Mena-Hernández et al. (2024) [43]	Randomized crossover (14/14 by sequence); 4-week washout	28 → 17 analyzed (8 withdrew, 3 excluded for low adherence; 39.3%)	Adherent completers	Ad libitum; photographic records incomplete, intake not estimated	Daily photo uploads and weekly calls; good adherence defined as >85% of days	Timing of adherence exclusions differs between text and flow diagram	Lower (attrition >20%, completers only)
Lowe et al. (2020), TREAT [44]	Randomized parallel (72/69)	141 → 105 completed (25.5%); CMT 23.6%, TRE 27.5%	ITT-labelled mixed model for weight (116 with data); DXA in a self-selected local subgroup ($n = 50$)	Ad libitum; intake not recorded	App surveys; 83.5% (TRE) and 92.1% (CMT) of responses adherent	Lean mass reported with a 99.7% CI and ALMI with a 95% CI	Moderate for weight; lower for lean mass (self-selected subgroup; multiple secondary outcomes)
Domaszewski et al. (2022) [45]	Reported randomized parallel (23/23); sequence generation and concealment NR	46 → 46 (no losses reported)	ANCOVA; ITT not stated	No target; intake not quantified	Food-diary calendar; all TRE participants reported success	CIs with signs inconsistent with the estimates; FFM significance differs between text and table; clock times ambiguous	Lower (inconsistent key data)
Sampieri et al. (2024) [41]	Randomized parallel, stratified; 8-h (10:00–18:00), 10-h, and 12-h windows centred on 14:00, and a normal diet	41 → 40 (2.4%); per-protocol 32	ITT-labelled analysis of 40; per-protocol analysis excluded 8 non-adherent participants	Habitual intake; 3-day diaries at baseline and week 8	Weekly calls; 3-day diaries (8 window failures detected)	No key inconsistency	Moderate (modified ITT; adherence sampled over 3 days)

Table S3 (continued)

Study [ref.]	Allocation	Participants: entered → analyzed (attrition); attrition by condition	Analysis population	Energy intake (prescribed, assessed, or controlled)	Adherence to the eating window	Consistency of reported data	Confidence (main reason)
**Studies with concurrent structured exercise training (Table 2)**							
Miladi et al. (2025; 2026 companion) [36, 35]	Reported randomized parallel, 4 groups; sequence generation and concealment NR	75 allocated → 61 (18.7%); ETRE-PA 25%, LTRE-PA 25%, LTRE 21%, control 0%	Completers	1200–1500 kcal/day stated in Methods but intake described elsewhere as ad libitum; intake not assessed	Method NR; exercise at the end of each window (16:00 vs. 20:00)	Allocated (75) vs. enrolled (61) numbers and control-group size (15 vs. 16) differ between sections	Lower (attrition gap >10 pp; inconsistent flow)
Yu and Ueda (2025), 8-week trial [46]	Randomized parallel (8/8/8); computer-generated sequence, sealed envelopes	24 → 24 (0%)	All randomized participants (linear mixed model)	Usual diet; protein ≥1.2 g/kg/day advised; 3-day photographic logs; energy intake not quantified	Daily calls/messages; 85.6% (eTRE), 89.5% (dTRE), 95.6% (control)	Adherence values differ between Results and Discussion	Moderate (intake not quantified; small groups)
Moro et al. (2020) [33]	Randomized parallel (8/8)	16 → 16 (no losses reported)	Reported sample; ITT not stated	Both arms prescribed 4800 kcal/day with menus; no food diary during the intervention	Dietitian supervision; adherence percentage NR	Baseline fat mass differs between table and text; IGF-1 direction differs; window end time differs	Lower (inconsistent key data)
Tovar et al. (2021); Richardson et al. (2023) [39, 38]	Randomized crossover (8 TRE-first, 7 ND-first among completers); self-selected window	19 → 15 (21.1%)	Complete pairs; mixed model with sequence term (sequence effect for body fat %)	Instructed to match first-arm intake; 3-day logs; 2513 vs. 2421 kcal/day ($p = 0.41$)	Self-selected window; adherence percentage NR	Regional pre/post means incompatible with reported changes (companion report); washout described differently	Lower (attrition >20%, complete pairs only)
Cui et al. (2025) [34]	Randomized parallel, 4 groups (13/16/15/14)	58 → 54 (6.9%); 0–13.3% by group	Completers	No target; intake not recorded	Meal photographs; adherence NR in main text; training 16:00–18:00, near or after window close	Dispersion in text and table inconsistent (SE vs. SD)	Lower (attrition gap >10 pp; completers)
Ameur et al. (2024) [50]	Randomized parallel, 3 groups (allocated 23/27/24)	74 → 64 (13.5%); 11.1–16.7% by group	Completers (ANCOVA)	Ad libitum; 7-day food diaries; energy intake decreased more with TRE-HIFT than HIFT	Daily messages; adherence NR; training at 17:00, after the window	FFM values incompatible with body weight and fat mass; randomized *n* given as 74 and 64	Lower (inconsistent key data)
Moro et al. (2016) [51]	Randomized parallel (17/17); outcome assessors blinded	34 → 34 (no losses reported)	Reported sample; ITT not stated	Habitual energy targeted; 7-day diaries; 2735 vs. 2910 kcal/day; protein ∼1.9 g/kg/day; whey after training	Weekly dietitian interviews; percentage NR; training 16:00–18:00, within the window	Fat-mass percentage change in text differs slightly from table means	Moderate (self-reported intake and adherence; small groups)
Kotarsky et al. (2021) [52]	Reported randomized parallel (10/13)	23 → 21 (8.7%); control 0%, TRE 15.4%	Completers	Ad libitum; 3-day food records; week 7: 1974 vs. 1806 kcal/day ($p = 0.510$); protein 0.8–0.9 g/kg/day	Sampled-day records; mean recorded window 7.0 h; training in the fed state	No key inconsistency	Lower (attrition gap >10 pp; completers)
Tinsley et al. (2017) [49]	Reported randomized parallel (about 14/14 starters); TRE on non-training days only	28 → 18 analyzed; withdrawal TRF 7.1%, ND 35.7%; 4 further exclusions	Restricted sample of 18 (missing values imputed within it)	Ad libitum; 4-day food records; intake about 667 kcal/day lower on TRF days	Daily checklists; 95.9% in the retained sample	Participant flow differs between 2015 conference abstract and full report (full report used)	Lower (attrition >20%; differential loss; adherence exclusions)
Tinsley et al. (2019) [47]	Randomized parallel (14/13/13), stratified; HMB double-blind	40 → 31 (22.5%); control 35.7%, TRF 7.7%, TRF+HMB 23.1%; per protocol 24	ITT ($n = 40$; likelihood-based mixed models); per protocol 24	Prescribed REE × 1.5 − 250 kcal/day; whey provided; weighed food records; intake below target	Diet records and questionnaires; eating-schedule compliance ≥89%	No key inconsistency; fat-mass advantage only in per-protocol analysis	Lower (attrition gap >10 pp)
Hofstätter et al. (2025) [42]	Reported randomized parallel, 3 groups; allocation counts NR	52 → 36 (30.8%; 15 withdrew, 1 excluded)	Completers meeting adherence criteria	Ad libitum; intake not quantified; 212-kcal snack before training in one group	Daily diaries; fasting compliance 89.4% and 92.1%	Negative standard deviations for fat-mass change; randomized *n* differs between Abstract and Methods	Lower (attrition >20%; inconsistent data)

Notes: Confidence refers to each study's between-condition findings (Section 2.6 of the main text). It was rated *lower* when allocation to the conditions being compared was non-randomized, when attrition exceeded 20% and only completers or a per-protocol sample were analyzed, when attrition differed by more than 10 percentage points between conditions, or when key outcome data were internally inconsistent; *moderate* when attrition exceeded 20% but all randomized participants were analyzed with likelihood-based or imputation methods, or when another domain was limited (for example, completers-only analysis with low attrition, or energy intake prescribed or not quantified); and *higher* otherwise. Attrition is calculated as (entered − analyzed)/entered and, where noted, includes exclusions from analysis. The appraisal was performed by one author (Z.Y.) against the full-text reports. ALMI, appendicular lean mass index; ANCOVA, analysis of covariance; BIA, bioelectrical impedance analysis; BMI, body mass index; CCR, continuous caloric restriction; CI, confidence interval; CMT, consistent meal timing; CR, caloric restriction; DXA, dual-energy X-ray absorptiometry; ER, energy restriction; FFM, fat-free mass; GEE, generalized estimating equations; HIFT, high-intensity functional training; HMB, β-hydroxy-β-methylbutyrate; IF, intermittent fasting; IGF-1, insulin-like growth factor 1; ITT, intention-to-treat; ND, normal diet; NR, not reported; PA, physical activity; PAL, physical activity level; pp, percentage points; REE, resting energy expenditure; RMR, resting metabolic rate; SD, standard deviation; SE, standard error; TRF, time-restricted feeding.
